# Mast cell degranulation is negatively regulated by the Munc13-4-binding small-guanosine triphosphatase Rab37

**DOI:** 10.1038/srep22539

**Published:** 2016-03-02

**Authors:** Hironori Higashio, Yoh-ichi Satoh, Tomoyuki Saino

**Affiliations:** 1 Department of Chemistry, Center for Liberal Arts and Sciences, 2-1-1 Nishitokuta, Yahaba, Iwate 028-3694, Japan; 2 Division of Cell Biology, Department of Anatomy, 2-1-1 Nishitokuta, Yahaba, Iwate 028-3694, Japan; 3 Department of Medical Education, Iwate Medical University, 2-1-1 Nishitokuta, Yahaba, Iwate 028-3694, Japan

## Abstract

Mast cell degranulation is regulated by the small guanosine triphosphatases (GTPases) Rab27a and Rab27b, which have distinct and opposing roles: Rab27b acts as a positive regulator through its effector protein Munc13-4, a non-neuronal isoform of the vesicle-priming Munc13 family of proteins, whereas Rab27a acts as a negative regulator through its effector protein melanophilin, by maintaining integrity of cortical filamentous actin (F-actin), a barrier to degranulation. Here we investigated the role of Rab37, one of the Rab GTPases assumed to be implicated in regulated secretion during mast cell degranulation. Using the RBL-2H3 mast cell line, we detected Rab37 on the secretory granules and found that antigen-induced degranulation was extensively increased by either knockdown of Rab37 or overexpression of a dominant-active Rab37 mutant. This hypersecretion phenotype in the Rab37-knockdown cells was suppressed by simultaneous knockdown of Rab27a and Rab27b or of Munc13-4, but not by disruption of cortical F-actin. We further found that Rab37 interacted with Munc13-4 in a GTP-independent manner and formed a Rab27-Munc13-4-Rab37 complex. These results suggest that Rab37 is a Munc13-4-binding protein that inhibits mast cell degranulation through its effector protein, by counteracting the vesicle-priming activity of the Rab27-Munc13-4 system.

Mast cells are granulated cells that play a central role in allergic responses such as anaphylaxis and asthma, as well as certain innate and adaptive immune responses^1^. Secretory granules of mast cells are lysosome-related organelles containing inflammatory mediators such as histamine, as well as lysosomal components including β-hexosaminidase and CD63^1^,^2^,^3^. Antigen-mediated cross-linking of the high-affinity IgE receptor FcεRI activates a complicated signalling cascade leading to the activation of protein kinase C (PKC), elevation of intracellular Ca^2+^ concentration^1^,^4^, and microtubule-dependent translocation of secretory granules towards the plasma membrane^5^,^6^, eventually resulting in the release of granule contents. This Ca^2+^ -dependent, regulated exocytosis in mast cells is called degranulation, which involves granule-granule and granule-plasma membrane fusion via multigranular or compound exocytosis^7^. This mechanism of regulated exocytosis is distinct from that of neurotransmitter release, where individual synaptic vesicles fuse with the plasma membrane; however, mast cell degranulation and neurotransmitter release generally share common protein families^8^. Mast cell degranulation involves membrane fusion driven by members of the soluble *N*-ethylmaleimide-sensitive fusion protein attachment protein receptor (SNARE) family and regulation of membrane fusion by various regulators such as small guanosine triphosphatases (GTPases) of the Rab family, Munc13-4 of the Munc13 family, Munc18-2 of the Munc18 family, proteins of the synaptotagmin family, complexin II of the complexin family, and Doc2α of the Doc2 family^8^.

The Rab family small GTPases are key regulators of intracellular membrane traffic in all eukaryotes. More than 60 Rab proteins have been identified in mammals: these proteins regulate vesicle formation from donor membranes, vesicle movement between organelles, and/or fusion of the vesicles to target membranes within the intracellular network of membrane traffic. Each Rab protein functions as a molecular switch by cycling spatiotemporally between the GTP-bound active form and the GDP-bound inactive form, coupled with association/dissociation to the membrane. Active Rab proteins recruit specific effectors to their respective membrane location, allowing each Rab-effector complex to play its part in regulating membrane traffic as previously described^9^. The Rab proteins implicated in (or possibly implicated in) regulated exocytosis are classified by amino acid sequence similarity and are referred to as secretory Rabs, composed of the Rab3 subfamily (Rab3a/b/c/d), the Rab27 subfamily (Rab27a/b), Rab37, and Rab26^10^. Mast cell degranulation involves three 3 secretory Rabs: Rab3d, Rab27a, and Rab27b. The first secretory Rab identified in mast cells was Rab3d, localised on secretory granules, and translocated towards the plasma membrane upon antigen stimulation^11^. Exogenous expression of Rab3d or the dominant-negative Rab3d mutant inhibits the antigen-induced release of granule content from the RBL-2H3 mast cell line^12^, implicating the role of Rab3d in mast cell degranulation. In contrast, GTP-induced degranulation proceeds normally in mouse peritoneal mast cells from Rab3d-deficient mice^13^, making the role of Rab3d somewhat confusing. The second and third secretory Rabs that were identified in mast cells are Rab27a and Rab27b, which share more than 70% amino acid identity^14^. Based on their intracellular localisation, they are thought to regulate the movement and/or exocytosis of dense-core vesicles and lysosome-related organelles in various non-neuronal secretory cells^15^,^16^. For example, Rab27a positively regulates exocytosis in cytotoxic T lymphocytes (CTLs)^17^, platelets^18^, and neutrophils^19^, whereas Rab27b does the same in platelets^20^ and neutrophils^21^. As Rab27a and Rab27b are often co-expressed in various secretory cells^16^ and share 11 common effector proteins^10^,^14^, the Rab27 family of proteins were originally thought to be functionally redundant^22^. Nonetheless, recent evidence indicates that Rab27a and Rab27b play distinct roles in regulated exocytosis in certain cell types, probably by recruiting different effector proteins^21^,^23^,^24^. In mouse bone marrow-derived mast cells (BMMCs), Rab27a negatively regulates degranulation by maintaining cortical filamentous-actin (F-actin) integrity, probably through interaction of its effector protein melanophilin with myosin Va, whereas Rab27b positively regulates degranulation, probably through its effector protein Munc13-4^23^, a non-neuronal isoform of vesicle-priming proteins of the Munc13 family^25^ that is known to regulate exocytosis in CTLs^26^, platelets^18^, neutrophils^19^,^27^, and mast cells^23^,^28^,^29^.

Rab37, a member of the secretory Rabs, was originally identified in BMMCs and the mouse MC-9 mast cell line: Rab37 is found on granular structures labelled by LysoTracker, a fluorescent dye used to stain lysosome-related organelles^30^. Although the role of Rab37 in mast cell degranulation remains unknown, Rab37 was recently shown to regulate exocytosis in some cell types. In insulinoma cell lines, Rab37 is expressed and localised on insulin-containing granules, while its knockdown impairs granule docking and subsequent fusion with the plasma membrane^31^. In macrophages, knockdown analyses show that Rab37 positively regulates lipopolysaccharide-induced release of tumour necrosis factor α possibly through its binding protein Munc13-1^32^, a member of vesicle-priming proteins of the Munc13 family, originally identified in neurons^25^. On the other hand, in human umbilical vein endothelial cells, Rab37 is expressed and localised on endothelium-specific organelles called Weibel–Palade bodies that contain bioactive molecules. However, Rab37-knockdown has little or no effect on Weibel–Palade body exocytosis^33^. Thus, the role of Rab37 in regulated exocytosis remains unclear. In this study, we investigated the regulatory role of Rab37 in mast cell degranulation and the functional relationship between Rab37 and the Rab27-Munc13-4 positive regulatory system.

## Results

### Rab37 expression and its intracellular localisation in the RBL-2H3 mast cell line

**Figure 1:**
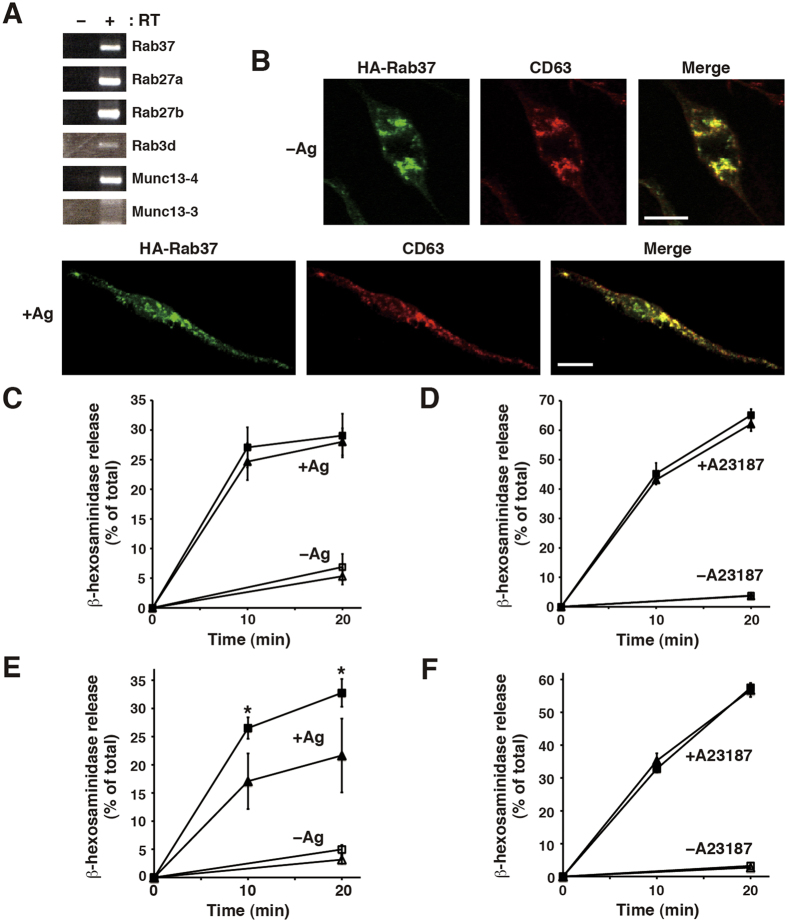
The Rab37 GTPase resides on secretory granules and its dominant-active mutant shows increased antigen-induced degranulation in RBL-2H3 cells. (**A**) Endogenous Rab37 expression. Total RNA from the cells was subjected to first-strand cDNA synthesis with (+) or without (−) reverse transcriptase (RT), followed by PCR amplification with specific primer sets for the indicated genes. PCR products were separated on agarose gel and stained with ethidium bromide. The full-length gel images are presented in Supplementary Fig. S1. (**B**) Intracellular localisation of Rab37. Cells transfected with the HA-Rab37 plasmid were sensitised with IgE and stimulated with (+Ag) or without (−Ag) antigen in a Ca^2+^ -free medium, followed by fixation and co-immunostaining with anti-HA and anti-CD63 antibodies. Merged images are also shown on the right. Note that panels (−Ag) are enlarged images of the cell focused on perinuclear region with parts of unstained rod-like protrusions and that panels (+Ag) are images of the whole cell. Bars, 15 μm. (**C**) Antigen-induced degranulation in the cells expressing exogenous Rab37. G418-surviving cells transfected with the HA-Rab37 (square, n = 4) or the control (triangle, n = 4) plasmid were sensitised with IgE and stimulated with (+Ag) or without (−Ag) antigen for the indicated time periods. (**D**) A23187/TPA-induced degranulation in the cells expressing exogenous Rab37. The G418-surviving transfectants described in (**C**) were stimulated with (+A23187) or without (−A23187) A23187 together with 12-*O*-tetradecanoylphorbol-13-acetate (TPA) for the indicated time periods. (**E**) Antigen-induced degranulation in the cells expressing a dominant-active Rab37 mutant. G418-surviving cells transfected with the HA-Rab37DA (square, n = 4) or the control (triangle, n = 4) plasmid were sensitised and stimulated as in (**C**). (**F**) A23187/TPA-induced degranulation in the cells expressing the dominant-active Rab37 mutant. The G418-surviving transfectants described in (**E**) were stimulated as in (**D**). In (**C–F**) β-hexosaminidase activity was measured in the supernatant and the cell lysate, and the degree of release was expressed as a percentage of the total activity. *p < 0.05, compared to the stimulated control transfectant.

Rab37 was originally identified in mouse BMMCs and the mouse MC-9 mast cell line^30^. To determine whether Rab37 is involved in mast cell degranulation, we first examined Rab37 expression in the rat mucosal mast cell line, RBL-2H3. Endogenous Rab37 protein could not be clearly detected by western blotting or immunofluorescence microscopy using our polyclonal antibody raised against rat Rab37 or the commercially available polyclonal antibody against human Rab37. Therefore, Rab37 expression in RBL-2H3 cells was assessed using reverse-transcriptase (RT)-polymerase chain reaction (PCR). As shown in Fig. 1A, PCR products of expected size were obtained using primer sets for Rab37 as well as for Rab27a, Rab27b, Rab3d, and Munc13-4 as positive controls^11^,^12^,^28^,^29^,^34^, but not for the neuron-specific Munc13-3 used as a negative control^25^, in an RT-dependent manner. These results indicate that Rab37 are endogenously expressed in RBL-2H3 cells.

In BMMCs, exogenously expressed Rab37 is found on granular structures labelled by LysoTracker, a fluorescent dye for staining acidic organelles, suggesting its localisation on secretory granules^30^. Therefore, we examined the intracellular localisation of haemagglutinin (HA)-tagged Rab37 (HA-Rab37) in RBL-2H3 cells by immunofluorescence microscopy using an anti-HA antibody together with an antibody against CD63, a secretory granule marker. Specificity of anti-HA antibody against the HA-Rab37 protein was confirmed by western blotting using a lysate from the transfected RBL-2H3 cells (Fig. S2). As shown in Fig. 1B, HA-Rab37 was found to be distributed in the perinuclear region and extensively overlapped with CD63 distribution (−Ag: Pearson’s correlation coefficient (r) = 0.91 ± 0.03, n = 11). As secretory granules are translocated to the cell periphery upon antigen stimulation^5^,^6^,^35^,^36^, we further examined the distribution of HA-Rab37 in stimulated RBL-2H3 cells. When HA-Rab37-expressing cells were stimulated with antigen in a Ca^2+^ -free medium (to prevent membrane fusion), HA-Rab37 and CD63 were translocated to the cell periphery including the rod-like protrusions (Fig. 1B,  + Ag: r = 0.77 ± 0.13, n = 10). Together, these results suggest that Rab37 is expressed and resides on secretory granules in RBL-2H3 cells, consistent with recent global study of Rab GTPases expressed in the cells reported by Azouz *et al*.^34^, in the course of our study.

### Effects of Rab37 overexpression on degranulation in RBL-2H3 cells

Release of β-hexosaminidase, a well-known marker of mast cell degranulation, represents the release of other mediators such as histamine^37^. Therefore, to examine whether Rab37 is involved in mast cell degranulation, we assessed the stimulus-induced release of β-hexosaminidase from RBL-2H3 cells expressing HA-Rab37. To overcome the low transfection efficiency of RBL-2H3 cells, we first developed a method to concentrate transfectants, using the antibiotic geneticin (G418) and plasmids carrying the G418-resistantance gene, Neo^r^. The cells were transfected with the plasmid carrying Neo^r^ and 24 hours later, were grown in medium containing 0.6 mg/ml G418 for an additional 24 h, after which, the surviving cells were analysed. When the cells were transfected with the enhanced green fluorescent protein (EGFP)-expression plasmid, flow cytometry revealed that G418 selection significantly increased the proportion of EGFP-positive cells (Fig. S3: from 53.2% to 89.2%). In addition, western blot analyses showed significant increase in the amount of HA-Munc13-4 in the lysate after G418 selection (Fig. S3), suggesting the efficient concentration of HA-Munc13-4-expressing cells. Since Munc13-4, when overexpressed, is known to enhance antigen-induced degranulation^28^,^36^, we further examined the effect of G418 selection on antigen-induced release of β-hexosaminidase using the cells transfected with the HA-Munc13-4-expression plasmid. Without G418 selection, we observed minimal increase in β-hexosaminidase release from the cells transfected with the HA-Munc13-4-expression plasmid under experimental conditions. Nevertheless, G418 selection revealed a marked increase in β-hexosaminidase release from the HA-Munc13-4 transfectants (Fig. S3). Thus, the 1-day G418 selection described above is a convenient and effective method to concentrate Neo^r^-carrying transfectants to up to 90% of a cellular population. This method can help clarify the function of various genes of interest in RBL-2H3 cells.

We then compared the degranulation profiles of G418-surviving cells transfected with the HA-Rab37-expression plasmid and cells with the control vector. As shown in Fig. 1C, upon antigen stimulation of the control transfectant, β-hexosaminidase release occurred within the first 10 min, followed by a slow residual release during the next 10 min (+Ag, closed triangles), whereas only a low-level spontaneous release (typically up to <3% of the total) was observed during a 20-min incubation without antigen stimulation (−Ag, open triangles). In the HA-Rab37 transfectant, both kinetics and amounts of the β-hexosaminidase release were comparable to those from the control transfectant (±Ag, open/closed squares). We also compared the profiles of degranulation triggered by Ca^2+^ influx and PKC activation using the Ca^2+^ ionophore A23187 and the phorbol ester 12-*O*-tetradecanoylphorbol-13-acetate (A23187/TPA stimulation) between the transfectants (Fig. 1D). In the control transfectant, the kinetics of the β-hexosaminidase release was similar to that during antigen stimulation as described above. Nonetheless, the A23187/TPA stimulation was much stronger in terms of β-hexosaminidase release compared to antigen stimulation. These exocytic profiles were comparable between HA-Rab37 and the control transfectants, indicating that the Rab37 overexpression has little or no effect on the stimulus-induced or spontaneous release of β-hexosaminidase from RBL-2H3 cells.

We further compared the degranulation profiles between the G418-surviving cells transfected with the expression plasmid for HA-tagged Rab37 mutant and the cells with the control vector. Western blotting showed that while both the HA-tagged dominant-active Rab37 mutant (HA-Rab37DA) mimicking a GTP-bound active form of Rab37 and the HA-tagged dominant-negative Rab37 mutant (HA-Rab37DN) mimicking a GDP-bound inactive form of Rab37 were extensively expressed in COS7 cells, only HA-Rab37DA was expressed in RBL-2H3 cells (Fig. S2). Immunofluorescence microscopy showed that HA-Rab37DA is co-localized with CD63 in RBL-2H3 cells (Fig. S2: r = 0.85 ± 0.04, n = 10), suggesting its secretory-granule localisation. As shown in Fig. 1E,F, while the profiles of A23187/TPA-induced (+A23187) and the spontaneous (−Ag and −A23187) β-hexosaminidase release were almost identical between the HA-Rab37DA and the control transfectants, antigen-induced (+Ag) release from the HA-Rab37DA transfectant (closed squares) was markedly higher than that from the control transfectant (closed triangles). These results suggest that Rab37 is involved in antigen-induced degranulation in RBL-2H3 cells, consistent with previously shown global analysis of the Rab GTPases expressed in the cells^34^.

### Effect of Rab37 knockdown on degranulation in RBL-2H3 cells

**Figure 2:**
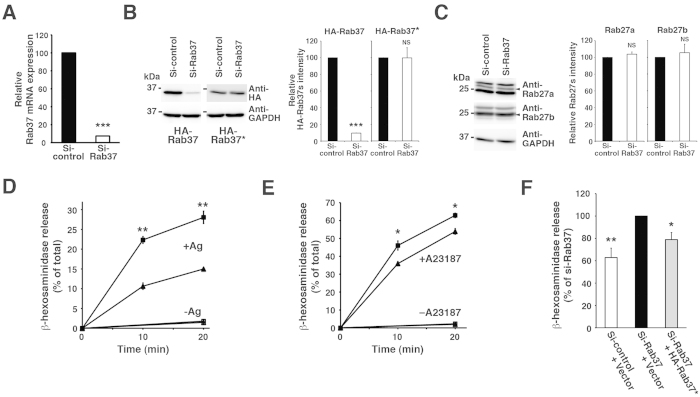
Hypersecretion phenotype of Rab37-knockdown RBL-2H3 cells. (**A**) Rab37 mRNA expression in the Si-Rab37 transfectant. The relative mRNA expression of Rab37 compared to GAPDH was determined in the G418-surviving cells transfected with Si-Rab37 or Si-control plasmid using quantitative real-time RT-PCR. The expression level of Rab37 mRNA in the Si-control transfectant was set to 100 and the relative expression level in the Si-Rab37 transfectant is presented as mean ± SD (n = 3). ***p < 0.001. (**B**,**C**) Specificity of the Rab37 knockdown. Cell lysates (20 μg) from the G418-surviving cells transfected with Si-control or Si-Rab37 plasmid (**C**), together with HA-Rab37 or the knockdown-resistant HA-Rab37* plasmid (**B**) were subjected to western blot analyses with the antibodies indicated. Arrowheads indicate position of Rab27a and Rab27b (**C**). The full-length blotting images of (**B,C**) are presented in Supplementary Figs S4 and S5, respectively. The graphs represent the relative quantity of each Rab, with the quantity in the Si-control transfectant set to 100. Bars represent mean ± SD (n = 4). ***p < 0.001; NS, not significant. (**D,E**) Degranulation in the Rab37-knockdown cells. G418-surviving cells transfected with Si-Rab37 (squares; n = 4) or Si-control (triangles; n = 4) plasmid were sensitised with IgE and stimulated with (+Ag) or without (−Ag) antigen (**D**) or directly stimulated with (+A23187) or without (−A23187) A23187/TPA (**E)** for the indicated time periods. The degree of β-hexosaminidase release was measured and presented as in Fig. 1(C-F). *p < 0.05; **p < 0.01, compared to the stimulated Si-control transfectant. (**F**) Rescue experiment using HA-Rab37*. The degree of 10-minute β-hexosaminidase release from the G418-surviving cells transfected with Si-Rab37 or Si-control plasmid, together with HA-Rab37* or control (Vector) plasmid, was examined as in (**D**). After subtraction of the spontaneous release, the antigen-induced release from the Si-Rab37 transfectant with the control plasmid was set to 100, and the relative release from other transfectants compared to the Si-Rab37 transfectant with the control plasmid is presented as mean ± SD (n = 4). *p < 0.05; **p < 0.01.

Next, we compared the degranulation profiles of G418-surviving RBL-2H3 cells transfected with either Rab37-silencing plasmid (Si-Rab37) or control vector (Si-control). Quantitative real-time RT-PCR analysis revealed that, in the Si-Rab37 transfectant, the expression level of Rab37 mRNA was reduced to <10% of that in the Si-control transfectant (Fig. 2A). The Si-Rab37 plasmid also efficiently reduced expression of the HA-Rab37 protein in RBL-2H3 cells without affecting expression levels of the knockdown-resistant HA-Rab37* (Fig. 2B) and the Rab27 subfamily proteins (Fig. 2C). As shown in Fig. 2D,E, both the antigen-induced (+Ag) and the A23187/TPA-induced (+A23187) β-hexosaminidase release from the Si-Rab37 transfectant (closed squares) were markedly higher than that from the Si-control transfectant (closed triangles), whereas profiles of spontaneous release (−Ag, −A23187) were almost identical. However, this hypersecretion phenotype was attenuated by the expression of HA-Rab37* (Fig. 2F). As granular content of CD63 and β-hexosaminidase appeared identical between the transfectants (Fig. S6), these results collectively suggest an inhibitory role of Rab37 in stimulus-induced degranulation in RBL-2H3 cells.

### Effect of cortical F-actin disassembly on degranulation in Rab37-knockdown RBL-2H3 cells

Cortical F-actin acts as a barrier for mast cell degranulation, thus preventing inappropriate and/or excess release of granule contents^38^. In BMMCs, Rab27a negatively regulates degranulation by maintaining cortical F-actin integrity, probably via interaction between its effector protein melanophilin and myosin Va^23^. To confirm the relationship between degranulation and integrity of cortical F-actin in RBL-2H3 cells, we first performed the β-hexosaminidase release assay with or without pretreatment of the cells with latrunculin B (LatB), an actin depolymerization reagent. Immunofluorescence microscopy showed that phallacidin staining of F-actin in the LatB-untreated cells extended along the plasma membrane, including the rod-like protrusions, but in the cells treated with 1 μM LatB, no F-actin staining was observed in addition to alterations in cell shape (Fig. S7). Under these conditions, antigen-induced and spontaneous release of β-hexosaminidase were both significantly higher in the LatB-treated cells than in the untreated cells (Fig. S7), indicating a barrier effect of cortical F-actin with respect to degranulation of RBL-2H3 cells.

**Figure 3:**
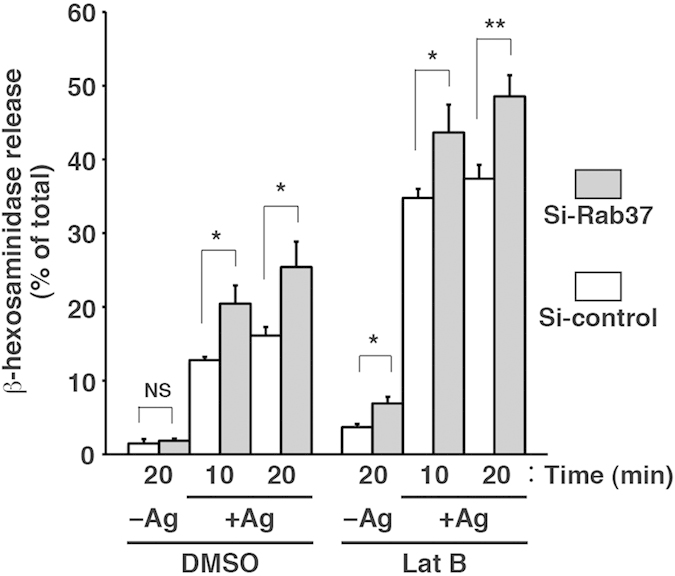
Rab37 knockdown increases the antigen-induced β-hexosaminidase release independent of cortical F-actin integrity. G418-surviving RBL-2H3 cells transfected with Si-Rab37 (grey bar, n = 4) or Si-control (white bar, n = 4) plasmid were sensitised with IgE, incubated with (LatB) or without (DMSO) LatB, and stimulated with (+Ag) or without (−Ag) antigen for the indicated time periods. The degree of β-hexosaminidase release was measured and presented as in Fig. 1(C−F). *p < 0.05; **p < 0.01; NS, not significant.

We then addressed the possibility that Rab37 negatively regulates degranulation of RBL-2H3 cells by maintaining cortical F-actin integrity, as is the case for Rab27a in BMMCs^23^. Immunofluorescence microscopy showed that regardless of antigen stimulation, both abundance and distribution of F-actin and CD63 were apparently indistinguishable between Si-Rab37 and Si-control transfectants (Fig. S8). To directly examine if cortical F-actin is required for the inhibitory effect of Rab37 on degranulation, we performed the β-hexosaminidase release assay using these transfectants with or without pretreatment with 1 μM LatB. The 1-μM LatB treatment was sufficient for almost complete disruption of F-actin and to achieve maximum increase in β-hexosaminidase release upon antigen stimulation (Fig. S7). As shown in Fig. 3, the Si-Rab37 transfectant still showed an increase in β-hexosaminidase release upon antigen stimulation compared to the Si-control transfectant, even when pretreated with LatB (+Ag, LatB), suggesting that Rab37 negatively regulates degranulation independently of cortical F-actin. In addition, the Si-Rab37 transfectant showed increased spontaneous release of β-hexosaminidase compared to the Si-control transfectant only when pretreated with LatB (Fig. 3, −Ag, LatB), suggesting that even in the absence of stimulation, Rab37 knockdown increases fusion-competent secretory granules, whose spontaneous exocytosis is usually prevented by a cortical F-actin barrier. Taken together, these results suggest that Rab37 negatively regulates mast cell degranulation by preventing secretory granules from becoming fusion-competent but not by maintaining cortical F-actin integrity.

### Functional relationship between Rab37 and the Rab27-Munc13-4 system

**Figure 4:**
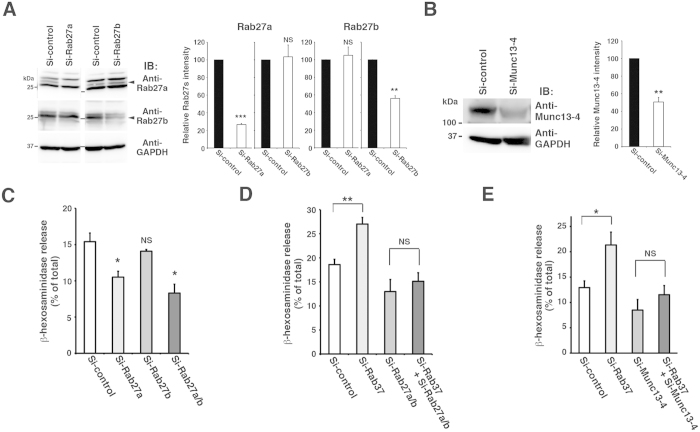
Hypersecretion phenotype of the Rab37-knockdown RBL-2H3 cells requires an intact Rab27-Munc13-4 system. (**A,B**) SiRNA-mediated downregulation of the Rab27 subfamily proteins (**A**) and Munc13-4 (**B**). Cell lysates (20 μg) from the G418-surviving cells transfected with Si-control plasmid together with Rab27a-specific siRNA (Si-Rab27a), Rab27b-specific siRNA (Si-Rab27b), Munc13-4-specific siRNA (Si-Munc13-4), or control siRNA (Si-control) were subjected to western blot analyses with the antibodies indicated. Arrowheads indicate position of the Rab27 subfamily proteins (**A**). Full-length blotting images of (**A**,**B**) are presented in Supplementary Figs. S10 and S12, respectively. The graphs represent the relative quantity of the Rab27 subfamily proteins (**A**) and Munc13-4 (**B**) with the quantity in the Si-control transfectant set to 100. Bars represent mean ± SD (n = 4). ***p < 0.001; **p < 0.01; NS, not significant. (**C**) Effects of Rab27 knockdown on antigen-induced degranulation. G418-surviving cells transfected with Si-control plasmid together with Si-control, Si-Rab27a, Si-Rab27b, or a combination of Si-Rab27a and Si-Rab27b (Si-Rab27a/b) were used. (**D,E**) Effect of Rab27 (**D**) or Munc13-4 (**E**) knockdown on antigen-induced degranulation in Rab37-knockdown cells. G418-surviving cells transfected with the following combinations were used: Si-control plasmid plus Si-control (Si-control), Si-Rab37 plasmid plus Si-control (Si-Rab37), Si-control plasmid plus Si-Rab27a/b (Si-Rab27a/b), Si-Rab37 plasmid plus Si-Rab27a/b (Si-Rab37 + Si-Rab27a/b), Si-control plasmid plus Si-Munc13-4 (Si-Munc13-4), or the Si-Rab37 plasmid plus Si-Munc13-4 (Si-Rab37 + Si-Munc13-4). In (**C**−**E**) the cells were sensitised and stimulated with or without antigen for 10 min. The degree of β-hexosaminidase release was measured as in Fig. 1(C−F) and the release without stimulation was subtracted. Error bars represent mean ± SD (n = 4). *p < 0.05; **p < 0.01; NS, not significant.

The Rab27 subfamily of proteins requires the effector protein Munc13-4 to drive mast cell degranulation^28^,^29^,^39^. To gain insights into the functional relationship between Rab37 and the Rab27-Munc13-4 system, the effect of downregulation of the Rab27 subfamily proteins or Munc13-4 on the hypersecretion phenotype of Rab37-knockdown cells was examined. We first confirmed expression levels of the Rab27 subfamily and Munc13-4 proteins in the G418-surviving cells transfected with Rab27a-specific siRNAs (including Si-Rab27a), Rab27b-specific siRNAs (including Si-Rab27b), Munc13-4-specific siRNAs (including Si-Munc13-4), or control siRNA (Si-control), together with the empty vector carrying the Neo^r^ gene (Fig. 4A,B and Fig. S9). We also confirmed the extent of knockdown of the Rab27 subfamily by quantitative real-time RT-PCR (Fig. S11). As shown in Fig. 4A, the knockdown efficiency was quite moderate (Rab27a ~ 70% reduction, Rab27b ~ 40% reduction), while anti-Rab27a and anti-Rab27b antibodies showed specific downregulation of Rab27a and Rab27b by their specific siRNAs. Western blotting also showed a moderate knockdown of Munc13-4 (~50% reduction) by Si-Munc13-4 (Fig. 4B). We then examined antigen-induced β-hexosaminidase release from these siRNA transfectants in RBL-2H3 cells. Unexpectedly, under the given experimental conditions, Si-Rab27a, but not Si-Rab27b, reduced the extent of the release (Fig. 4C), seemingly contradicting previous observations in BMMCs that Rab27b has a positive regulatory role in degranulation^23^,^40^. While this discrepancy could not be resolved, a combination of Si-Rab27a and Si-Rab27b (Si-Rab27a/b) could reduce the extent of release more efficiently, consistent with the overlapping role of Rab27 subfamily proteins observed in degranulation in BMMCs^23^,^40^. When Rab37 knockdown was combined with a double-knockdown of Rab27a and Rab27b (Si-Rab37 + Si-Rab27a/b), the increase in β-hexosaminidase release caused by Rab37 knockdown was significantly attenuated (Fig. 4D). Moreover, similar attenuation was observed when Rab37 knockdown was combined with Munc13-4 knockdown (Fig. 4E, Si-Rab37 + Si-Munc13-4). Together, these results suggest that Rab37 is involved in the Rab27-Munc13-4-mediated degranulation pathway in mast cells, exerting an inhibitory role.

### Physical interaction between Rab37 and Munc13-4

**Figure 5:**
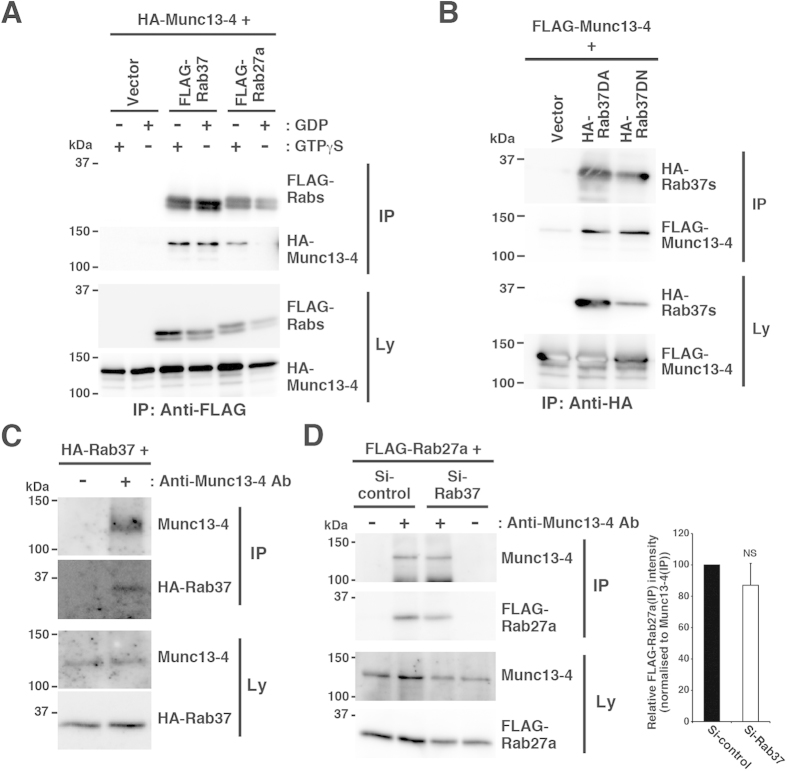
Rab37 interacts with Munc13-4. (**A**) Rab37-Munc13-4 interaction in COS7 cells. Lysates were prepared from the cells transfected with HA-Munc13-4 plasmid together with control (Vector), FLAG-Rab37, or FLAG-Rab27a plasmid in the presence (+) or absence (−) of GTPγS or GDP and subjected to immunoprecipitation with anti-FLAG antibody. The lysates (Ly) and immunoprecipitates (IP) were then subjected to western blot analyses with anti-HA (3F10) or anti-FLAG antibody. (**B**) Munc13-4 interacts with both the dominant active and the dominant negative mutants of Rab37. Lysates were prepared from COS7 cells transfected with FLAG-Munc13-4 plasmid together with control (Vector), HA-Rab37DA, or HA-Rab37DN plasmid and subjected to immunoprecipitation with anti-HA antibody. The lysates (Ly) and immunoprecipitates (IP) were analysed as in (**A**). (**C**) Rab37-Munc13-4 interaction in RBL-2H3 cells. Cells were transfected with the HA-Rab37 plasmid and cultured in the presence of G418 for 24 h. The surviving cells were treated with dithiobis succinimidyl propionate (DSP), lysed, and subjected to immunoprecipitation with (+) or without (−) anti-Munc13-4 antibody (Anti-Munc13-4 Ab). The lysates (Ly) and immunoprecipitates (IP) were then subjected to western blot analyses with anti-HA (3F10) or anti-Munc13-4 antibody. (**D**) Rab27-Munc13-4 interaction in the Rab37-knockdown RBL-2H3 cells. Cells were transfected with FLAG-Rab27a plasmid, together with Si-control or Si-Rab37 plasmid and cultured in the presence of G418 for 24 h. The surviving cells were treated with DSP, lysed in the presence of GTPγS, and subjected to immunoprecipitation with (+) or without (−) anti-Munc13-4 antibody (Anti-Munc13-4 Ab). The lysates (Ly) and immunoprecipitates (IP) were then subjected to western blot analyses with anti-FLAG or anti-Munc13-4 antibody. The graph represents the relative quantity of FLAG-Rab27a co-immunoprecipitated with Munc13-4, with the quantity in the Si-control transfectant set to 100. Bars represent mean ± SD (n = 4). NS, not significant. The full-length blotting images of (**A–D**) are presented in Supplementary Figs S13, S14, S16 and S17, respectively.

In RBL-2H3 cells, Rab37 seemed to be involved in the Rab27-Munc13-4-mediated degranulation pathway, raising alternative explanations: Rab37 could be directly implicated in the Rab27-Munc13-4-dependent process (i.e. vesicle priming) in the degranulation pathway or Rab37 may regulate some process upstream or downstream of the Rab27-Munc13-4-dependent process. To elucidate this process and the molecular mechanism that Rab37 is involved in, we attempted to identify Rab37 effector proteins via biochemical approaches. Because of the localisation of Rab37 on secretory granules, as is the case for Munc13-4^28^,^29^ and because of the interaction between Rab37 and Munc13-1 in macrophages^32^, we first tested the interaction between Rab37 and Munc13-4 by co-immunoprecipitation analyses. As shown in Fig. 5A, in the analyses using lysates from COS7 cells transfected with the HA-Munc13-4 plasmid, together with the expression plasmid for the FLAG-tagged version of Rab37 or Rab27a, HA-Munc13-4 was co-precipitated with FLAG-Rab37 in a GTP-independent manner. On the other hand, co-precipitation of HA-Munc13-4 with FLAG-Rab27a was observed only in the presence of guanosine 5′-[γ-thio] triphosphate (GTPγS), a non-hydrolysable GTP analogue (Fig. 5A). In addition, both HA-Rab37DA and HA-Rab37DN precipitated with FLAG-Munc13-4 (Fig. 5B), further supporting the GTP-independent interaction between Rab37 and Munc13-4. The Rab37-Munc13-4 and Rab27a-Munc13-4 co-precipitations were also captured by analyses using lysate from the transfected RBL-2H3 cells pretreated with dithiobis succinimidyl propionate (DSP), a cleavable crosslinker, indicating that these proteins indeed interacted within the cells (Fig. S15). In addition, both of the co-precipitations were unaffected by antigen stimulation (Fig. S15). We further found that HA-Rab37 was co-precipitated with endogenous Munc13-4 in cell lysates from transfected RBL-2H3 cells pretreated with DSP (Fig. 5C), strongly suggesting their specific and physiological interaction. To address the possibility that Rab37 modulates the Rab27-Munc13-4 interaction, we then examined the FLAG-Rab27a-endogenous Munc13-4 interaction in Rab37-knockdown RBL-2H3 cells. Co-immunopricipitation of the proteins was apparently not affected by Rab37 knockdown (Fig. 5D), suggesting that Rab37 does not modulate Rab27-Munc13-4 interaction.

### Details of the Rab37-Munc13-4 interaction

**Figure 6:**
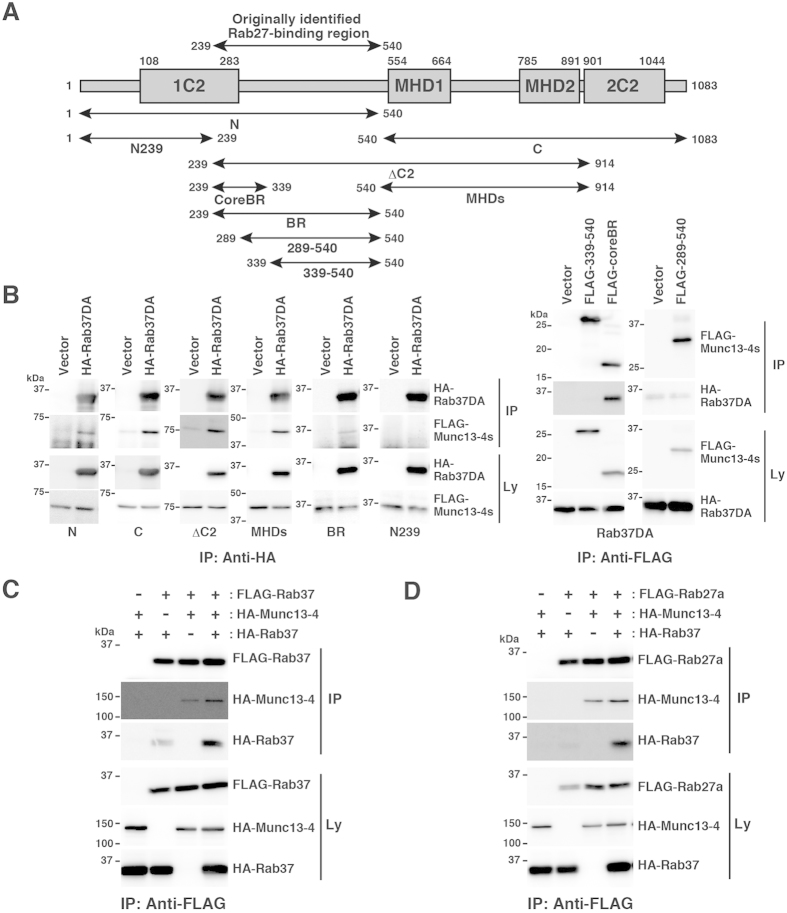
Analysis of the Rab37-Munc13-4 interaction. (**A**) Schematic representation of mutant Munc13-4 constructs. Domain organisation of Munc13-4 and amino acid positions corresponding to the truncation boundaries of each construct are shown. 1C2: the first Ca^2+^ -binding C2 domain, 2C2: the second Ca^2+^ -binding C2 domain, MHD1: the first Munc13 homology domain, and MHD2: the second Munc13 homology domain. (**B**) Identification of Rab37-interacting regions in Munc13-4 protein. Lysates from COS7 cells transfected with HA-Rab37DA or control (Vector) plasmid, together with the expression plasmid for FLAG-tagged Munc13-4 mutant protein presented in (**A**) were subjected to immunoprecipitation with anti-HA or anti-FLAG antibody as indicated. The lysates (Ly) and immunoprecipitates (IP) were subjected to western blot analyses with anti-HA (3F10) or anti-FLAG antibody. (**C**) Multiple Rab37 proteins interact with Munc13-4. COS7 cells were transfected with (+) or without (−) the following plasmids as indicated: FLAG-Rab37, HA-Munc13-4, and HA-Rab37 plasmids. The transfected cells were then lysed and subjected to immunoprecipitation with anti-FLAG antibody. The lysates (Ly) and immunoprecipitates (IP) were then analysed as in (**B**). (**D**) Formation of a Rab27-Munc13-4-Rab37 complex. COS7 cells were transfected with (+) or without (−) the following plasmids as indicated: FLAG-Rab27a, HA-Munc13-4, and HA-Rab37 plasmids. The transfected cells were then lysed in the presence of GTPγS and subjected to immunoprecipitation with anti-FLAG antibody. The lysates (Ly) and immunoprecipitates (IP) were then analysed as in (**B**). The full-length blotting images of (**B**−**D**) are presented in Supplementary Figs. S18, S20, and S21, respectively.

We examined the Rab37-Munc13-4 interaction by co-immunoprecipitation analyses using lysates from COS7 cells expressing HA-Rab37DA, together with the FLAG-tagged Munc13-4 mutant shown in Fig. 6A. These truncated Munc13-4 constructs were designed corresponding to the truncated Munc13-1 constructs previously described^41^, based on the conserved domain structure of the Munc13 protein family^25^. As shown in Fig. 6B, HA-Rab37DA was co-precipitated with the FLAG-tagged Munc13-4 mutants N, C, ΔC2, MHDs, BR, and CoreBR, but not with the N239, 339–540, and 289–540 mutants. This suggests that Munc13-4 has two Rab37-binding regions, MHDs (540–914) and the 239–289 region in coreBR, where the fragment containing either region can independently interact with HA-Rab37 in a GTP-independent manner (Fig. S19). Moreover, the anti-FLAG antibody immunoprecipitated FLAG-Rab37 together with HA-Munc13-4 and HA-Rab37 in lysates from the transfected COS7 cells (Fig. 6C), suggesting that Munc13-4 can interact with multiple Rab37 proteins. Next, we addressed the relationship between Rab37 and Rab27 with regard to binding with Munc13-4. As shown in Fig. 6D, the anti-FLAG antibody immunoprecipitated FLAG-Rab27a together with HA-Munc13-4 and HA-Rab37 in the analyses with lysates from the transfected COS7 cells prepared in the presence of GTPγS, suggesting that Rab37 can interact with the Rab27-Munc13-4 complex. We further found that the increasing concentration of Rab27a did not interfere with the Rab37-coreBR interaction (Fig. S22) and vice versa (Fig. S23), and that the ternary complex was formed by Rab37, Rab27a, and coreBR (Fig. S24). These results suggest that Rab37 is a Munc13-4-binding protein that negatively regulates mast cell degranulation without affecting the Rab27-Munc13-4 interaction.

## Discussion

In this study, we identified Rab37 to be a Munc13-4-binding protein on secretory granules that negatively regulates mast cell degranulation. Knockdown of Rab37 significantly enhanced antigen-induced degranulation in RBL-2H3 cells (Fig. 2D). Antigen-mediated cross-linking of FcεRI triggers a series of complicated signalling events leading to granule translocation towards the plasma membrane, PKC activation, and Ca^2+^ influx, and the latter two are necessary for activation of the molecular machinery directly driving secretory granule exocytosis^1^,^4^,^42^. Thus, degranulation can be directly induced by the A23187/TPA stimulation. Hypersecretion was still observed in Rab37-knockdown cells stimulated with A23187/TPA (Fig. 2E), suggesting that Rab37 is a negative regulator of mast cell degranulation, directly modulating the exocytic machinery rather than modulating other components such as FcεRI-triggered signalling or granule translocation. With respect to hypersecretion, we also showed that overexpression of Rab37DA, but not Rab37, enhanced antigen-induced degranulation (Fig. 1C,E), consistent with previous observations^34^. The phenotype of the Rab37 transfectant can be explained by saturation of the protein. In contrast, it is difficult to interpret the hypersecretion phenotype of the Rab37DA transfectant. Because Rab37DA is the non-functional Rab37 fixed in a GTP-bound form, Rab37DA may interfere with the negative regulatory role of endogenous Rab37 in degranulation by competing for correct subcellular localisation and/or by scavenging and sequestrating the effector protein from endogenous Rab37, as mentioned similarly^34^. However, A23187/TPA-induced hypersecretion was not observed in the Rab37DA transfectant (Fig. 1F). A possible explanation is that non-physiological potent activation may overcome Rab37DA-mediated moderate potentiation of degranulation observed in response to physiological activation, as is the case for degranulation in BMMCs from *ashen* mice^23^. Azouz *et al*. raised an alternative possibility that under A23187/TPA stimulation, Rab37DA cannot interfere with the negative regulatory role of endogenous Rab37 on degranulation, based on the observation that EGFP-Rab37DA is specifically sequestrated in the nucleus under the stimulation^34^. While the nature of the hypersecretion phenotype of the Rab37DA transfectant is currently unknown, our knockdown studies (Fig. 2D,E) suggest that Rab37 is a negative regulator of both antigen-induced and A23187/TPA-induced mast cell degranulation. In contrast, Rab37 positively regulates stimulus-induced exocytosis in insulinoma cells and macrophages^31^,^32^, which may be explained by the differences in the type and machinery of exocytosis in these secretory cell types (e.g. multivesicular versus sequential exocytosis).

Regulated exocytosis is generally subdivided into the following processes: vesicle docking, priming, and fusion^43^,^44^, mediated by SNAREs and/or various regulatory proteins, including Rabs. The process that Rab37 controls within the machinery of mast cell degranulation can be explained as follows. In mast cells, Rab27 positively regulates degranulation via interaction with its effector protein Munc13-4^23^,^28^,^29^,^39^. Knockdown analyses in this study revealed that Rab37 requires intact Rab27-Munc13-4 system for its negative regulatory role in degranulation (Fig. 4D,E), suggesting that Rab37 negatively regulates some process in the exocytic machinery upstream, downstream, or at the stage of the Rab27-Munc13-4-dependent priming process. The last notion is most plausible because the interaction between Rab37 and Munc13-4 (Fig. 5A–C) as well as formation of the Rab27-Munc13-4-Rab37 complex (Fig. 6D) were demonstrated by immunoprecipitation. In macrophages, Munc13-1 has also been identified as a Rab37-binding protein^32^. Furthermore, total internal reflection fluorescence microscopy studies have revealed that Rab37 in macrophages is translocated beneath the plasma membrane upon stimulation^32^, and that Rab37 knockdown in insulinoma cells reduces both, the number of secretory vesicles localised close to the plasma membrane and the frequency of fusion events^31^, suggesting a role of Rab37 in the docking process. As docking and priming were recently suggested to be closely linked processes^45^,^46^, these observations collectively suggest that Rab37 regulates stimulus-induced exocytosis at the stage of Munc13-dependent docking/priming.

The effector proteins for Rab37 in mast cells as well as in insulinoma cells are currently unknown^31^. Therefore, the precise modes of action of Rab37 during regulated exocytosis in these cells remain to be determined. On the other hand, degranulation assays using LatB point to a role of Rab37 in the exocytic machinery of mast cells. LatB treatment can abolish the barrier effect by cortical F-actin on mast cell degranulation^38^. After LatB treatment, Rab37 knockdown enhanced not only antigen-induced degranulation but also spontaneous exocytosis (Fig. 3), suggesting that Rab37 prevents both, excess generation of fusion-competent granules upon antigen stimulation and inappropriate generation of fusion-competent granules without stimulation. Thus, Rab37 may prevent excess and/or inappropriate granule docking/priming driven by the Rab27-Munc13-4 system by means of its unidentified effector protein. However, we cannot exclude the possibility of the involvement of Rab37 in certain F-actin-related processes, as the hypersecretion phenotype of the Rab37DA transfectant has been shown to be attenuated by cytochalasin D, an actin polymerization inhibitor^34^.

Immunoprecipitation analyses revealed that Rab37 interacted independently with the coreBR and the MHDs regions of Munc13-4 in a GTP-independent manner (Fig. 6B,C and S19). Moreover, we found that Rab37 and Rab27 could simultaneously interact with the coreBR region (Fig. S24) and that expression levels of Rab37 did not affect the Rab27-Munc13-4 interaction (Fig. 5D and Fig. S23). Together with the observation that the hypersecretion phenotype was caused by both, Rab37 knockdown and overexpression of the Munc13-4-interacting Rab37DA protein (Figs 1E and 2D,E) and that Rab37-Munc13-4 as well as Rab27-Munc13-4 interactions were unaffected by antigen stimulation (Fig. S15), these results collectively suggest that the priming activity of the Rab27-Munc13-4 complex is independent of interaction and dissociation of Rab37. The question of which negative regulatory mechanism Rab37 is engaged in can be addressed as follows. MHD1 and MHD2 are carboxy-terminal, conserved regions among the vesicle-priming proteins of the Munc13 family^25^. In Munc13-1, the region containing MHD1 and MHD2 at each end is a large part of the MUN domain, an autonomously folded minimal region responsible for synaptic-vesicle priming^41^,^47^, known to interact with SNAREs and SNARE complexes^41^,^48^,^49^,^50^,^51^. Furthermore, the MUN domain of Munc13-1 is known to interact with the SNARE syntaxin 1 and to promote its conformational change that allows for subsequent formation of the *trans*-SNARE complex responsible for fusion, the nature of vesicle priming^51^. Similar to Munc13-1, Munc13-4 is believed to function in docking/priming of lysosome-related secretory granules in some secretory cells of haematopoietic origin^26^,^28^,^39^,^52^. Thus, it is possible that, in mast cells, the Rab37 proteins bound to Munc13-4 recruit an unknown effector protein to counteract the Rab27-Munc13-4-mediated docking/priming activity via either downregulation of the docking/priming activity itself or some other anti-docking/priming function (e.g. interference with SNARE pairing or unwinding of the SNARE complex). Further studies of Rab37 effector proteins will help elucidate the molecular mechanisms of Rab37-mediated tuning of mast cell degranulation.

## Methods

### Cell culture and transfection

COS7 (obtained from RIKEN Cell Bank) and RBL-2H3 (obtained from American Type Culture Collection) cells were grown in Dulbecco’s modified Eagle’s medium (DMEM; Nacalai Tesque) containing 10% foetal bovine serum (Hyclone Laboratories), 50 U/ml penicillin, and 50 μg/ml streptomycin (Thermo Fischer Scientific) at 37 °C and 5% CO_2_. COS7 cells (4 × 10^5^ cells) were transfected with 0.5–2 μg per plasmid using Lipofectamine 2000 transfection reagent (Thermo Fischer Scientific), and RBL-2H3 cells (6 × 10^6^ cells) were electroporated in 600 μl DMEM with 25–50 μg per plasmid and/or with 200 nM siRNA at 250 V and 950 μF, using the Gene Pulser Xcell device (Bio-Rad). To concentrate the RBL-2H3 transfectant, 24 h after electroporation, G418/geneticin (InvivoGen) was added to the medium to the final concentration of 0.6 mg/ml and the culture was continued for additional 24 h. Thereafter, 48 hours after electroporation, the surviving cells were subjected to subsequent analyses.

### RT-PCR and quantitative real-time RT-PCR

Total RNA (1 μg) isolated from RBL-2H3 cells using the RNeasy Mini Kit (QIAGEN) was reverse-transcribed using the PrimescriptII High Fidelity RT-PCR Kit (TaKaRa) according to the manufacturer’s instructions. The expression of (potential) degranulation-related genes was then assessed by PCR with the primer sets listed in Supplementary Table S1 using the resulting first-strand cDNA as a template. A total of 35 cycles were performed, with denaturation at 98 °C for 1 min, annealing at 58 °C for 1 min, and extension at 68 °C for 1 min.

Quantitative real-time PCR analyses were performed using the SYBR Premix Ex Taq II (TaKaRa) and ABI7500 Real-time PCR system (Thermo Fischer Scientific) according to the manufacturer’s instructions. A total of 40 cycles were performed with denaturation at 95 °C for 5 s and annealing/extension at 60 °C for 34 s. The relative expression levels of Rabs compared to GAPDH were calculated by the standard-curve method using the Sequence Detection Software, version 1.4 (Thermo Fischer Scientific). Primer sets for the analyses were listed in Supplementary Table S2.

### cDNA cloning and plasmid construction

The EGFP-expressing vector pEGFP-N1 (the EGFP plasmid) and the siRNA expression vector pBAsi-mU6neo (the Si-control plasmid) were purchased from Clontech and TaKaRa, respectively; the mammalian expression vectors pCIneomyc, pCIneo3HA, and pCIneo3FLAG have been previously described^36^. Rat Rab37 cDNA was obtained by PCR using the following primer set, with the first-strand cDNA from RBL-2H3 cells used as a template: 5′-AGAGAGGAATTCATGACTGGCACACCAGGAGCTGCT-3′ and 5′-AGAGAGGCGGCCGCTCAGGAGTCACGCAAAGGAGCAGC-3′. After digestion, the resulting PCR product was subcloned into the expression vectors to produce HA-Rab37 and FLAG-Rab37 expression plasmids. cDNAs of the dominant-active (DA; Q89L) and the dominant-negative (DN; T43N) Rab37 mutants were then obtained via PCR-based mutagenesis and subcloned to produce the HA-Rab37DA and HA-Rab37DN expression plasmids, respectively. Rat Rab27a cDNA was obtained by PCR using the first-strand cDNA from RBL-2H3 cells as a template and subcloned to produce HA-Rab27a, FLAG-Rab27a, and Myc-Rab27a expression plasmids. cDNA of the DA (Q78L) Rab27a mutant were obtained via PCR-based mutagenesis and subcloned to produce FLAG-Rab27aDA and Myc-Rab27aDA plasmids. Mouse Munc13-4 (GenBank/EMBL/DDBJ accession No. NM_001009573) cDNA was obtained by PCR using the first-strand cDNA from mouse BMMCs^36^ as a template and subcloned to produce the HA-Munc13-4 expression plasmid. cDNAs of the truncation mutants of Munc13-4 presented in Fig. 6A and Supplementary Figure S19 were obtained by PCR and subcloned to produce the expression plasmids for FLAG-tagged Munc13-4 mutant proteins.

Using Invitrogen BLOCK-iT RNAi Designer (http://rnaidesigner.lifetechnologies.com/rnaiexpress/), we obtained the following target sequence for a rat Rab37 knockdown without affecting cell growth and viability: 5′-CCTGTTTCCTGATCCAATT-3′. The DNA fragment containing this sequence in both the sense and antisense directions was obtained and subcloned into the Si-control plasmid to produce the Si-Rab37 expression plasmid according to the manufacturer’s instructions. To obtain a cDNA of knockdown-resistant rat Rab37 (Rab37*), the target sequence in the Rab37 cDNA was changed to 5′-CATGCTTTCTGATTCAGTT-3′ by PCR-based site-directed mutagenesis and the resulting Rab37* cDNA was subcloned to produce HA-Rab37* expression plasmid. All plasmids used in this study carried the G418 resistance gene, Neo^r^, which allows for concentration of the RBL-2H3 transfectants.

### RNA interference

Stealth siRNA Duplexes and the Stealth RNAi Negative Control Medium GC Duplex were purchased from Thermo Fischer Scientific. All siRNA sequences used in this study are listed in Supplementary Table S3.

### Fluorescence and immunofluorescence microscopy

Immunostaining of the resting and stimulated RBL-2H3 cells was performed as previously described^36^. In some cases, disruption of the F-actin network was performed by incubation of cells in medium containing 1 μM LatB (Sigma-Aldrich) for 15 min prior to antigen stimulation or fixation. For staining of F-actin, the cells were incubated with 5 U/ml BODIPY FL PhallAcidin (Thermo Fischer Scientific) in PBS for 1 h after fixation, permeabilisation, and blocking. For immunostaining, Alexa488-conjugated goat anti-rat IgG and Alexa594-conjugated goat anti-mouse IgG antibodies (Thermo Fischer Scientific) were used as secondary antibodies. Stained cells were irradiated with a blue-beam (488 nm) from Argon ion laser and with a green-beam (543 nm) from He/Ne laser, and fluorescent images were acquired using a LSM510 meta/ConfoCor2 system (Carl Zeiss) equipped with the ×63 oil/1.4 NA (Fig. 1B, −Ag) and ×40 water/1.2 NA objectives (others), and with the FITC/Rhodamine (505–530 nm, > 560 nm) filter set. Co-localisation analyses were performed using Image J (Fiji). All images shown in this study are representative of at least 10 stained cells.

### Immunoprecipitation and western blotting

Before cell lysate preparation, the transfected RBL-2H3 cells were washed with PBS, treated with or without 1 mM DSP for 30 min, and quenched in 50 mM Tris-HCl, pH 7.5 for 20 min, at room temperature. Moreover, in some cases, the transfected RBL-2H3 cells were sensitised and stimulated as in the immunostaining procedure described previously^36^. The transfected COS7 or RBL-2H3 cells were then washed twice with PBS and lysed at 4 °C in lysis buffer (25 mM Tris-HCl, pH 7.5, 125 mM NaCl, 1 mM MgCl_2_, 1% Triton X-100, 1/100 vol. of protease inhibitor cocktail [Sigma-Aldrich, cat. #P8340]) or lysis buffer containing either 0.1 mM GDP or GTPγS (Sigma-Aldrich). After removing cell debris by centrifugation at 17 400 × ***g*** for 30 min, the lysates were incubated for 2 h at 4 °C with protein G-Sepharose beads (GE Healthcare) to remove the proteins bound to the beads, and the resulting cleared lysates were incubated at 4 °C overnight with either of the following mouse monoclonal antibodies attached to the beads: anti-HA (clone 12CA5; Roche Diagnostics), anti-FLAG (clone M2; Sigma-Aldrich), and anti-Myc (clone 9E10; Roche Diagnostics). The beads were washed 4 times with each lysis buffer and boiled in sodium dodecyl sulphate-polyacrylamide gel electrophoresis (SDS-PAGE) sample buffer (50 mM Tris-HCl, pH 6.8, 1% SDS, 10% glycerol, and 0.01% bromophenol blue) containing 0.1 M dithiothreitol. Western blotting was performed as previously described^36^. The following antibodies were used for western blotting: anti-FLAG (M2), anti-HA (clone 3F10; Roche Diagnostics), rabbit polyclonal anti-Rab27a (Sigma-Aldrich, cat. #R4655), anti-Rab27b (Immuno-Biological Laboratories, cat. #18973), anti-Munc13-4 (Santa Cruz Biotechnology, cat. #sc-50465), and anti-Rab37 (Assaybiotech, cat. #C18247), horse raddish peroxidase (HRP)-conjugated mouse monoclonal anti-FLAG (clone M2; Sigma-Aldrich), anti-GAPDH (clone 5A12; Wako), and anti-Myc (clone 9E10; Wako) served as the primary antibodies. HRP-conjugated AffiniPure goat anti-rat IgG L-chain specific, goat anti-mouse IgG L-chain specific, and F(ab′)_2_ fragment goat anti-rabbit IgG (H + L) (Jackson ImmunoResearch Laboratories) served as the secondary antibodies. Visualisation of immunoreactive proteins by chemiluminescence and quantification of the band intensity normalised to internal control were performed using a luminescent image analyser LAS3000 (GE Healthcare) according to the manufacturer’s instructions. All images presented in this study are representative of 3–4 independent experiments.

### β-hexosaminidase release assay

Assays for β-hexosaminidase release were performed as previously described^36^ with the following modifications. Transfected RBL-2H3 cells (1.25 × 10^5^ cells) were sensitised and stimulated with antigen in a final volume of 0.75 ml. Alternatively, the cells were directly stimulated with 1 μM A23187 (Calbiochem) together with 10 nM TPA (Sigma-Aldrich). In some cases, disruption of F-actin was performed as described above prior to antigen stimulation. Absorbance at 405 nm was measured using the Multiskan GO microplate spectrophotometer (Thermo Fischer Scientific). All data presented in this study are representative of 3–4 independent assays.

### Flow cytometry

The transfected RBL-2H3 cells were trypsinised, fixed with 2% paraformaldehyde in PBS for 15 min, and washed with PBS. The fixed cells were permeabilised with 0.025% Triton X-100 in PBS for 15 min and washed with PBS. After blocking with fluorescence-activated cell sorting (FACS) buffer (PBS containing 1% bovine serum albumin and 0.2% sodium azide) for 1 h, the permeabilised cells were incubated with anti-CD63 (clone AD1; BD Biosciences) antibody for 1 h, washed with FACS buffer, and incubated with an Alexa594-conjugated goat anti-mouse IgG antibody for 1 h. After washing with FACS buffer, the immunostained cells were analysed using a FACSCalibur flow cytometer (BD Biosciences). In the case of the EGFP-expressing cells, the cells were trypsinised, washed, resuspended in FACS buffer, and directly analysed by flow cytometry.

### Statistical analysis

All numerical data presented in this manuscript are mean ± standard deviation (SD) from 3−4 experiments. Statistical significance was then determined by two-tailed Student’s *t* test.

## Additional Information

**How to cite this article**: Higashio, H. *et al*. Mast cell degranulation is negatively regulated by the Munc13-4-binding small-guanosine triphosphatase Rab37. *Sci. Rep*. **6**, 22539; doi: 10.1038/srep22539 (2016).

## Supplementary Material

Supplementary Information
