# Supplementary material for: Mast cell degranulation is negatively regulated by the Munc13-4-binding small-guanosine triphosphatase Rab37
Source: Sci Rep. 2016 Mar 2;6:22539. doi: 10.1038/srep22539 (PMC4773767; doi:10.1038/srep22539)
Supplement: Supplementary Information [file srep22539-s1.pdf]

Supplementary Information

Mast cell degranulation is negatively regulated by the Munc13-4-binding small-guanosine triphosphatase Rab37

Hironori Higashio[*1], Yoh-ichi Satoh[2,3], and Tomoyuki Saino[2]

[1]Department of Chemistry, Center for Liberal Arts and Sciences, [2]Division of Cell Biology, Department of Anatomy, and [3]Department of Medical Education, Iwate Medical University, 2-1-1 Nishitokuta, Yahaba, Iwate 028-3694, Japan.

[*] To whom correspondence should be addressed:

Hironori Higashio

Department of Chemistry, Center for Liberal Arts and Sciences, Iwate Medical University, 2-1-1 Nishitokuta, Yahaba, Iwate 028-3694, Japan.

Tel.: +81-19-651-5110 (ext. 5037)

Fax: +81-19-698-1839

E-mail: higashio.hironori01@bs.naist.jp

Fig. S1

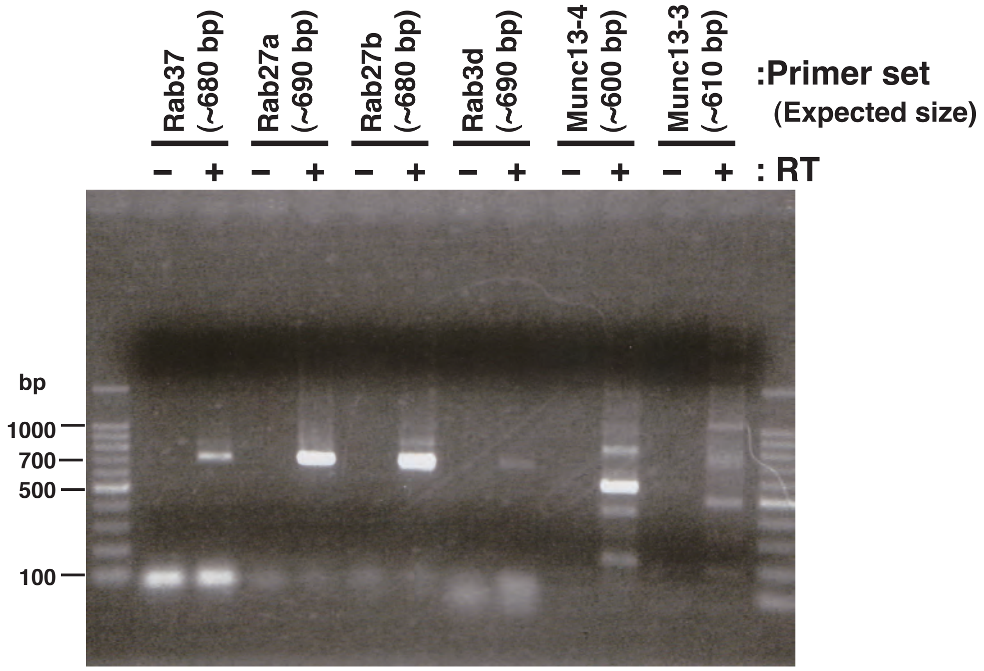
Rab37 (~680 bp)
Rab27a (~690 bp)
Rab27b (~680 bp)
Rab3d (~690 bp)
Munc13-4 (~600 bp)
Munc13-3 (~610 bp)
:Primer set
(Expected size)
− + − + − + − + − + − + : RT
bp
1000
700
500
100

Fig. S2

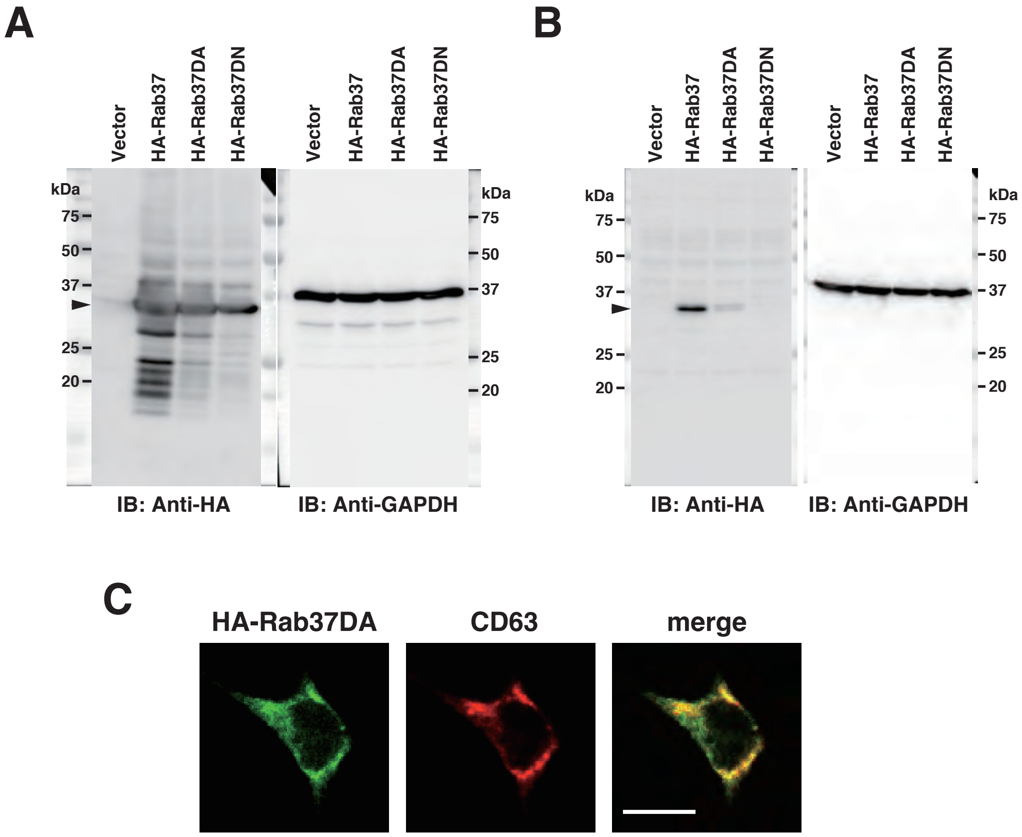
A
B
C
Vector
HA-Rab37
HA-Rab37DA
HA-Rab37DN
kDa
75
50
37
25
20
IB: Anti-HA
IB: Anti-GAPDH
HA-Rab37DA
CD63
merge

Fig. S3

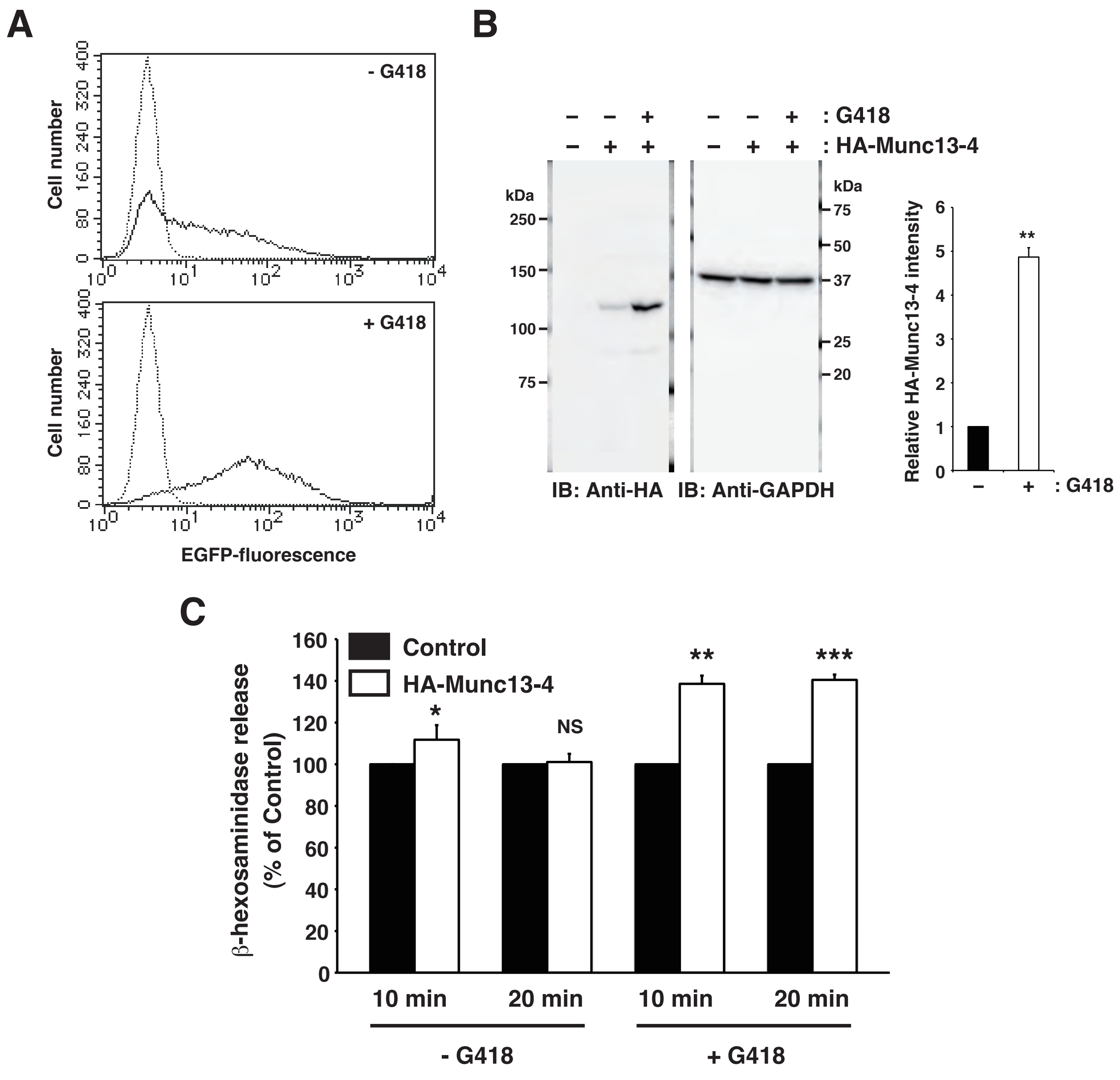
A
- G418
+ G418
Cell number
EGFP-fluorescence
B
: G418
: HA-Munc13-4
kDa
250
150
100
75
37
IB: Anti-HA
IB: Anti-GAPDH
Relative HA-Munc13-4 intensity
**
C
Control
HA-Munc13-4
β-hexosaminidase release (% of Control)
*
NS
**
***
10 min
20 min
- G418
+ G418

Fig. S4

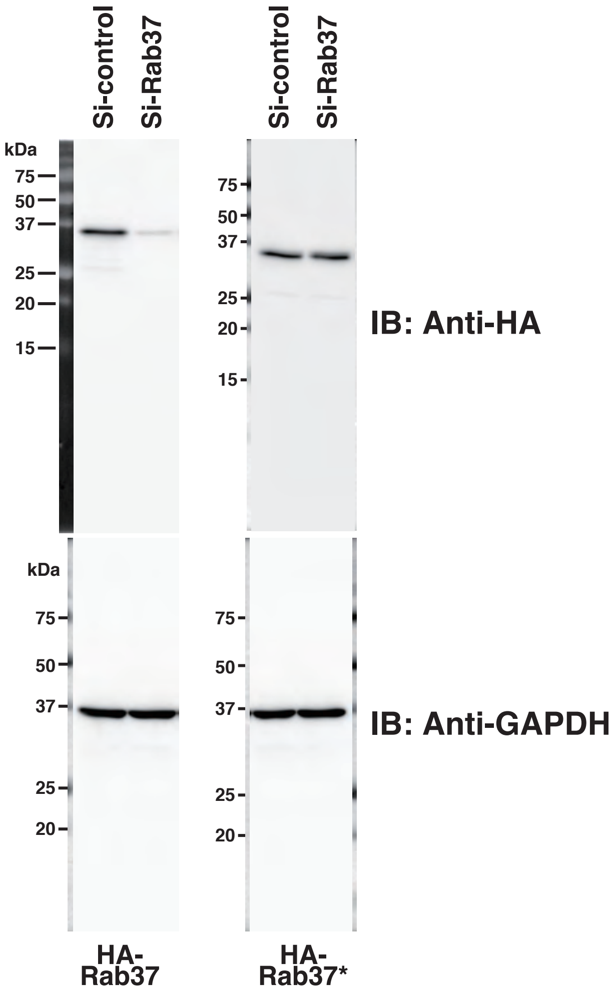

Si-control
Si-Rab37
Si-control
Si-Rab37
kDa
75
50
37
25
20
15
IB: Anti-HA
kDa
75
50
37
25
20
IB: Anti-GAPDH
HA-Rab37
HA-Rab37*

Fig. S5

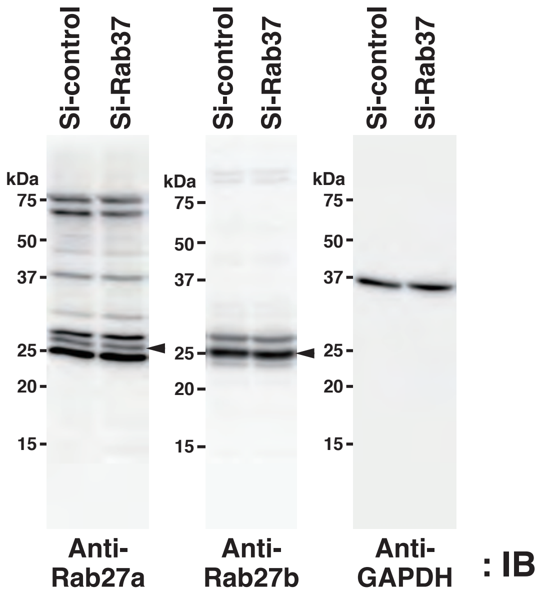
Si-control
Si-Rab37
Si-control
Si-Rab37
Si-control
Si-Rab37
kDa
75
50
37
25
20
15
kDa
75
50
37
25
20
15
kDa
75
50
37
25
20
15
Anti-
Rab27a
Anti-
Rab27b
Anti-
GAPDH
: IB

Fig. S6

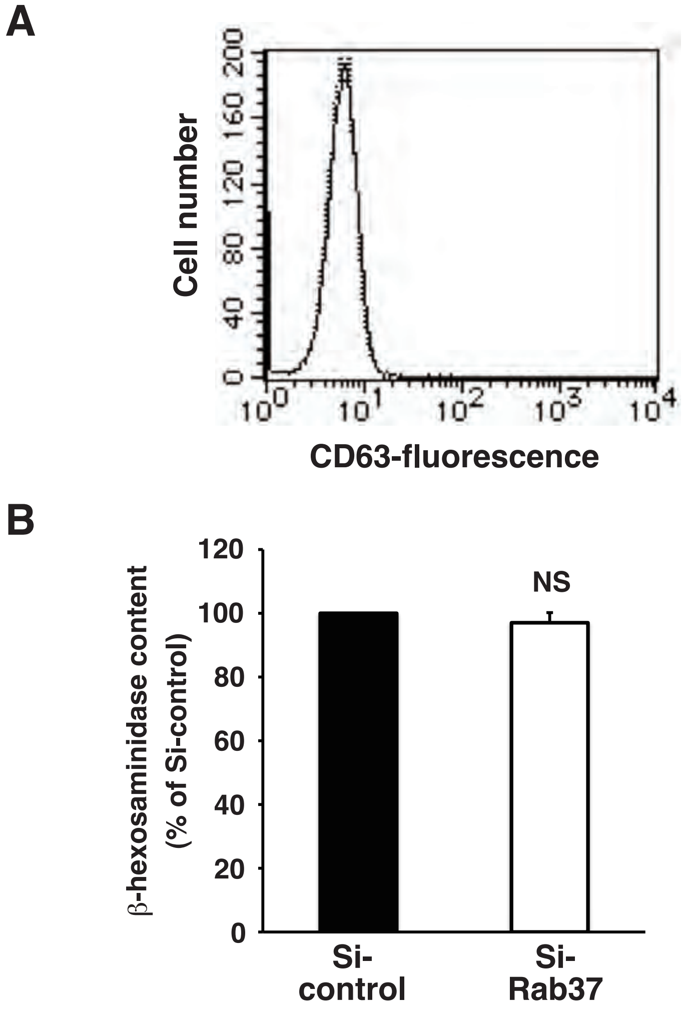
A
Cell number
0
40
80
120
160
200
10^0
10^1
10^2
10^3
10^4
CD63-fluorescence
B
β-hexosaminidase content
(% of Si-control)
0
20
40
60
80
100
120
NS
Si-
control
Si-
Rab37

Fig. S7

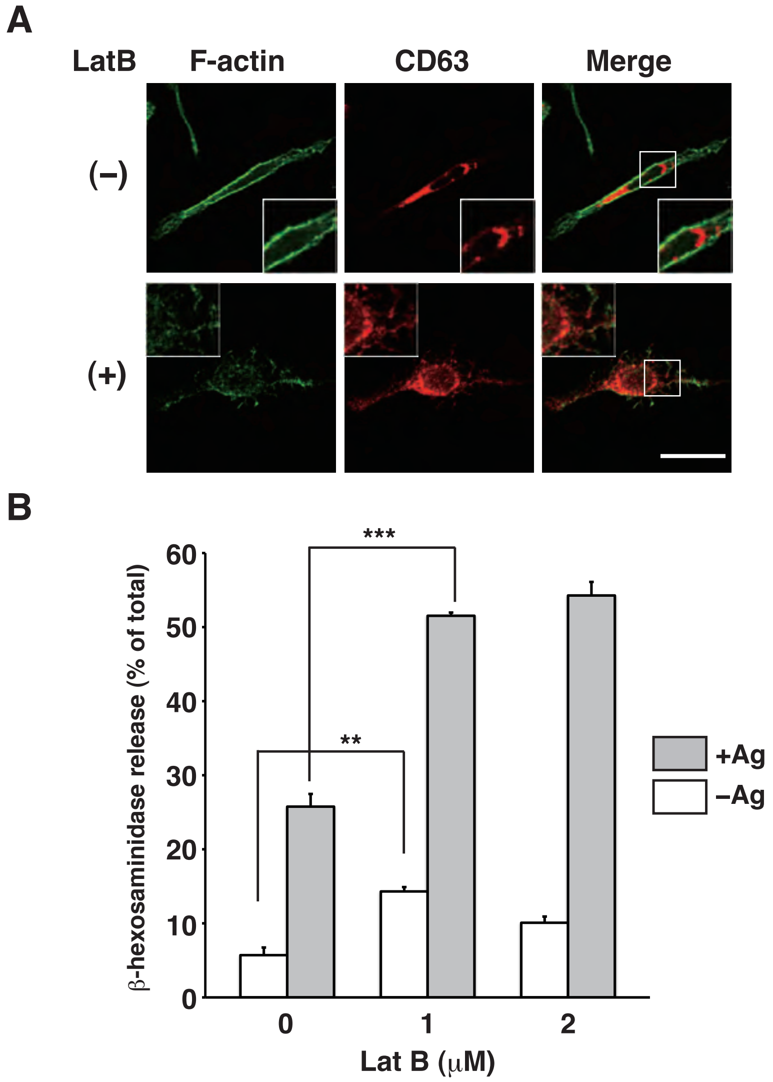
A
LatB
F-actin
CD63
Merge
(–)
(+)
B
***
**
60
50
40
30
20
10
0
β-hexosaminidase release (% of total)
0
1
2
Lat B (μM)
+Ag
–Ag

Fig. S8

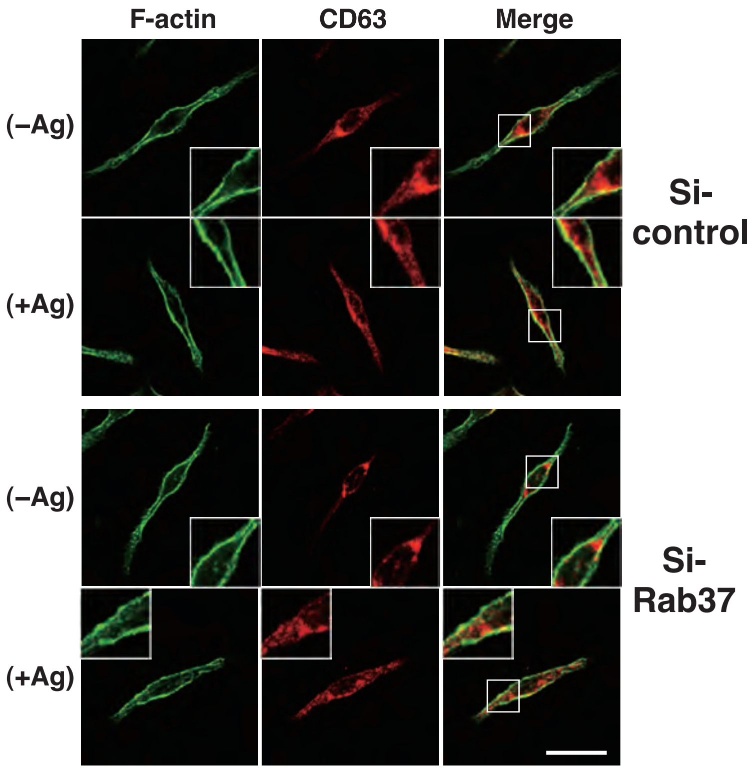

Fig. S9

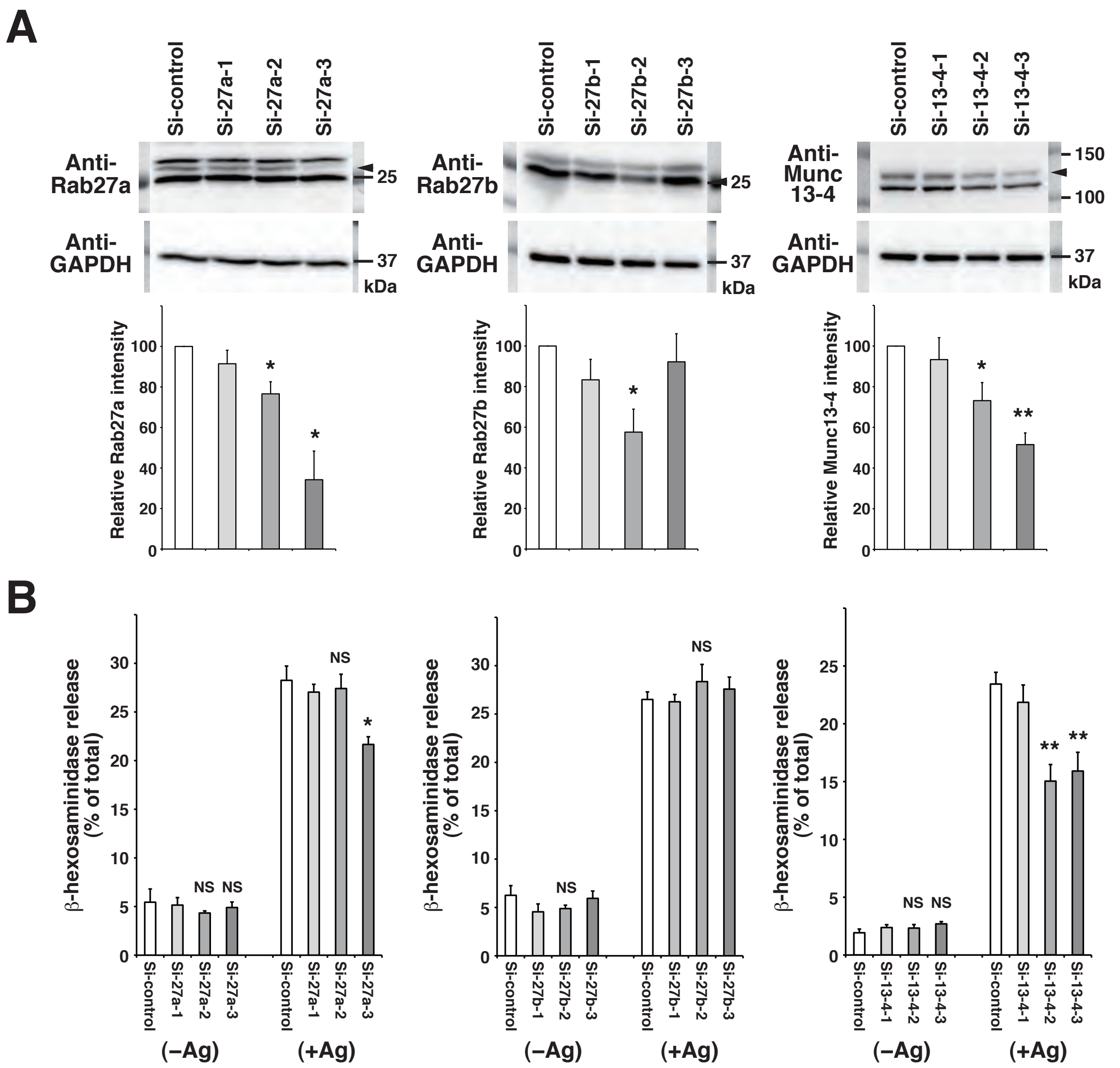
A
Si-control
Si-27a-1
Si-27a-2
Si-27a-3
Anti-Rab27a
Anti-GAPDH
25
37
kDa
Si-27b-1
Si-27b-2
Si-27b-3
Anti-Rab27b
Si-13-4-1
Si-13-4-2
Si-13-4-3
Anti-Munc 13-4
150
100
Relative Rab27a intensity
Relative Rab27b intensity
Relative Munc13-4 intensity
B
β-hexosaminidase release (% of total)
NS
(−Ag)
(+Ag)

Fig. S10

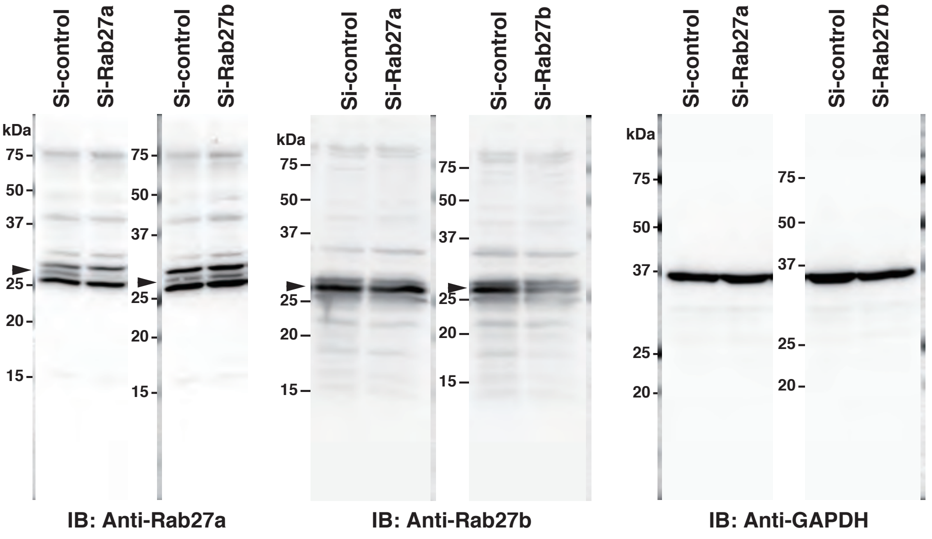

Si-control
Si-Rab27a
Si-control
Si-Rab27b
kDa
75
50
37
25
20
15
IB: Anti-Rab27a
IB: Anti-Rab27b
IB: Anti-GAPDH

Fig. S11

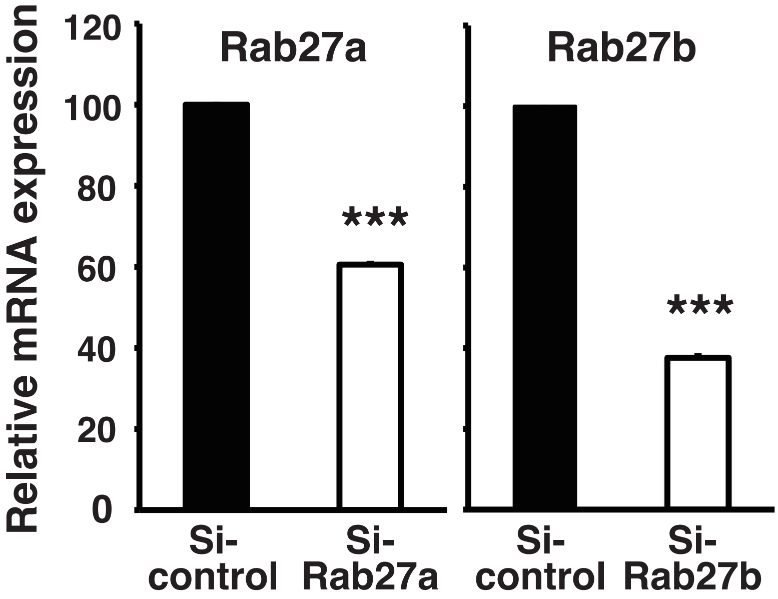
Rab27a
Rab27b
Relative mRNA expression
120
100
80
60
40
20
0
***
***
Si-
control
Si-
Rab27a
Si-
control
Si-
Rab27b

Fig. S12

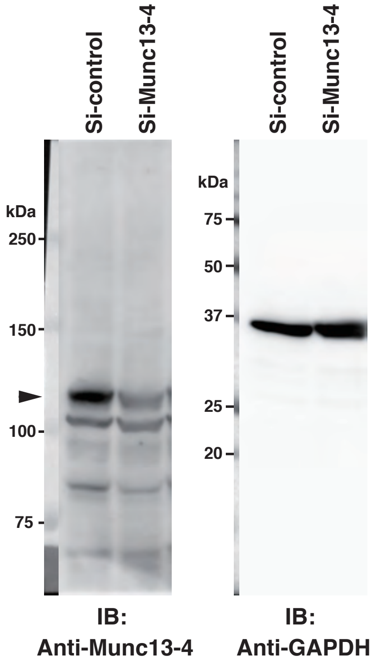
Si-control
Si-Munc13-4
kDa
250
150
100
75
IB:
Anti-Munc13-4
Si-control
Si-Munc13-4
kDa
75
50
37
25
20
IB:
Anti-GAPDH

Fig. S13

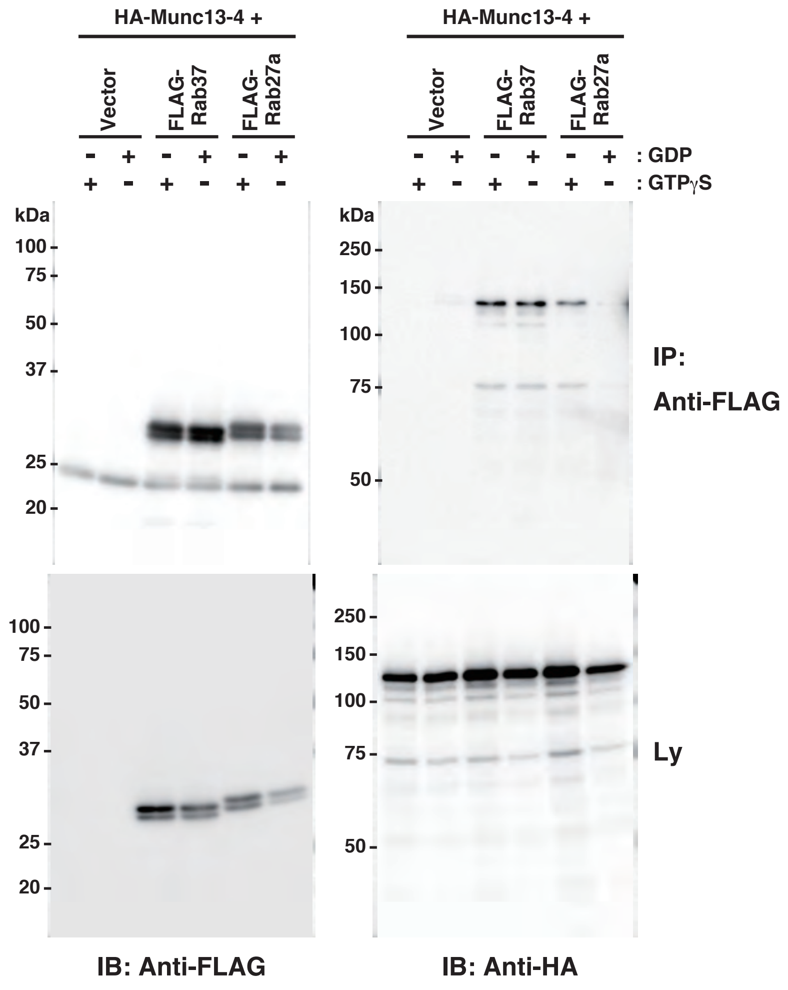
HA-Munc13-4 +
Vector
FLAG-Rab37
FLAG-Rab27a
: GDP
: GTPγS
kDa
100
75
50
37
25
20
250
150
IP: Anti-FLAG
Ly
IB: Anti-FLAG
IB: Anti-HA

Fig. S14

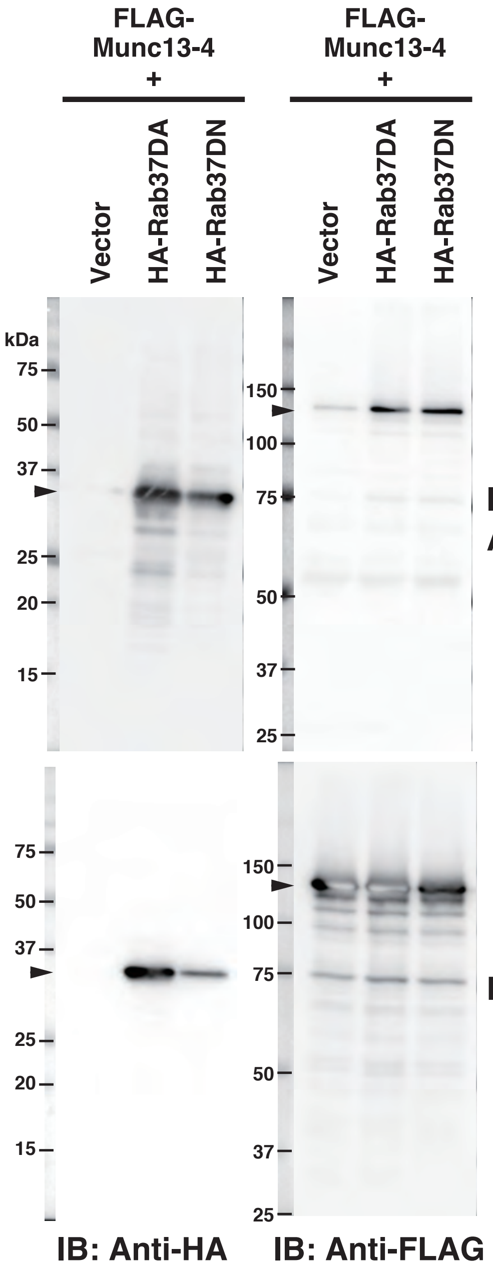

FLAG-
Munc13-4
+
Vector
HA-Rab37DA
HA-Rab37DN
kDa
75
50
37
25
20
15
150
100
75
50
37
25
IP:
Anti-HA
Ly
IB: Anti-HA
IB: Anti-FLAG

Fig. S15

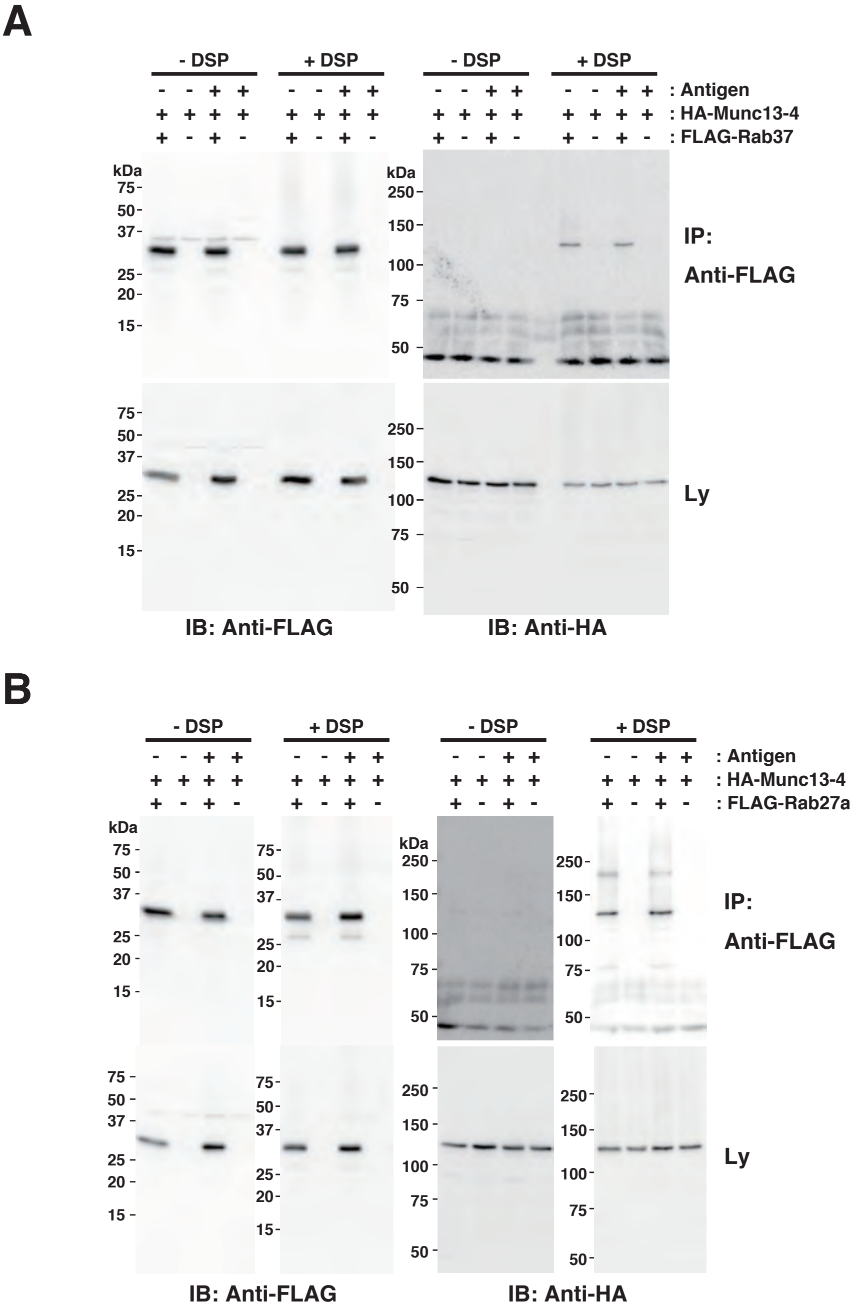
A
- DSP
+ DSP
- DSP
+ DSP
: Antigen
: HA-Munc13-4
: FLAG-Rab37
kDa
IP:
Anti-FLAG
Ly
IB: Anti-FLAG
IB: Anti-HA
B
- DSP
+ DSP
- DSP
+ DSP
: Antigen
: HA-Munc13-4
: FLAG-Rab27a
kDa
IP:
Anti-FLAG
Ly
IB: Anti-FLAG
IB: Anti-HA

Fig. S16

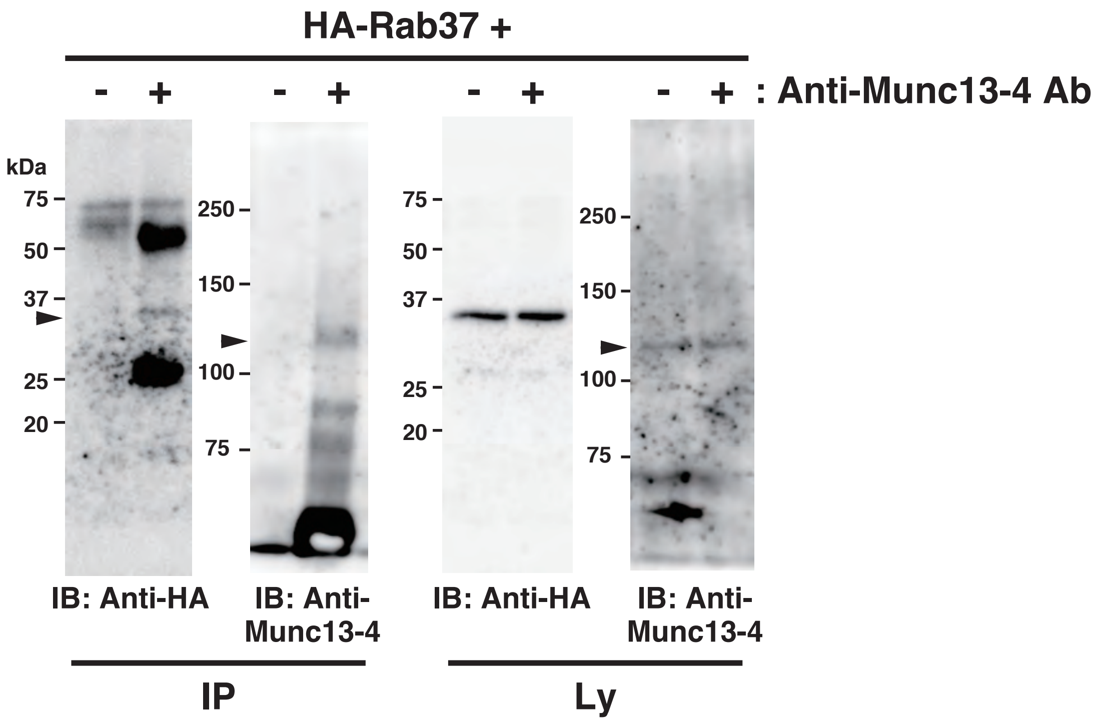
HA-Rab37 +
- + - + - + - + : Anti-Munc13-4 Ab
kDa
75
50
37
25
20
250
150
100
75
75
50
37
25
20
250
150
100
75
IB: Anti-HA
IB: Anti-Munc13-4
IB: Anti-HA
IB: Anti-Munc13-4
IP
Ly

Fig. S17

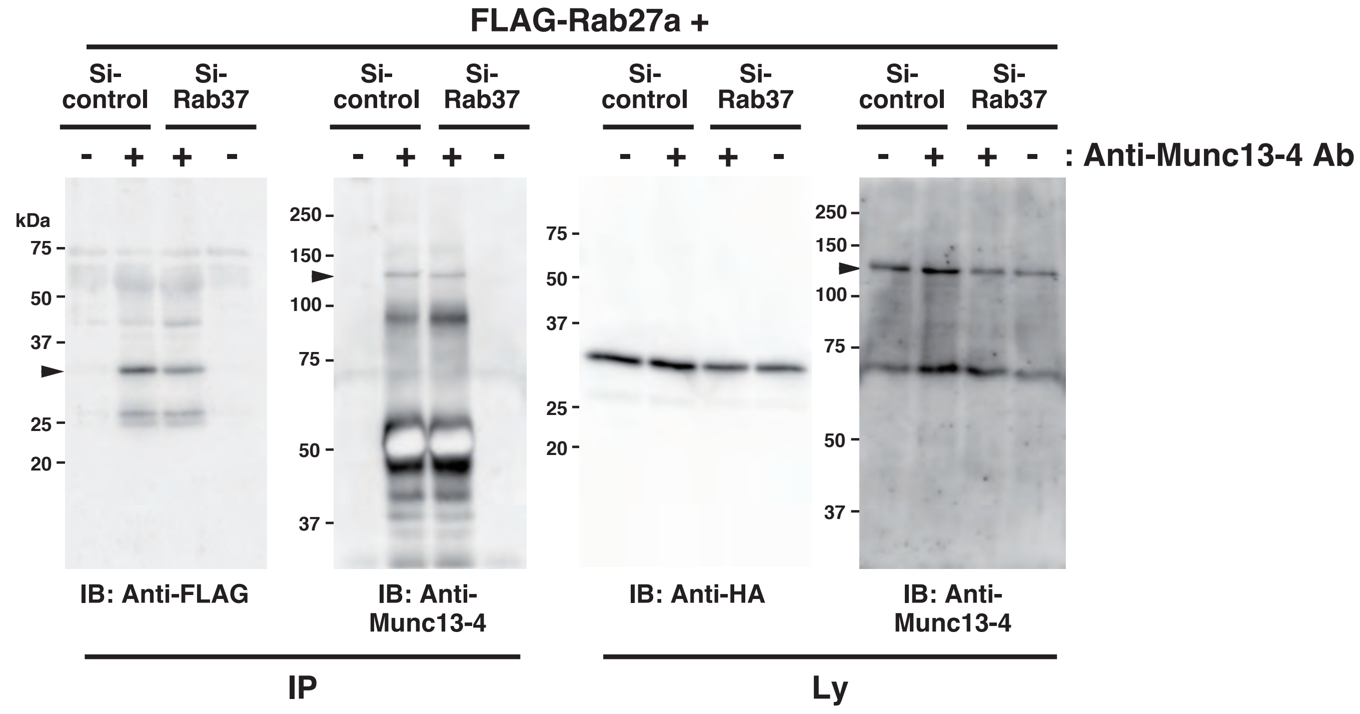
FLAG-Rab27a +
Si-control
Si-Rab37
- + + - : Anti-Munc13-4 Ab
kDa
75
50
37
25
20
250
150
100
IB: Anti-FLAG
IB: Anti-Munc13-4
IB: Anti-HA
IB: Anti-Munc13-4
IP
Ly

Fig. S18

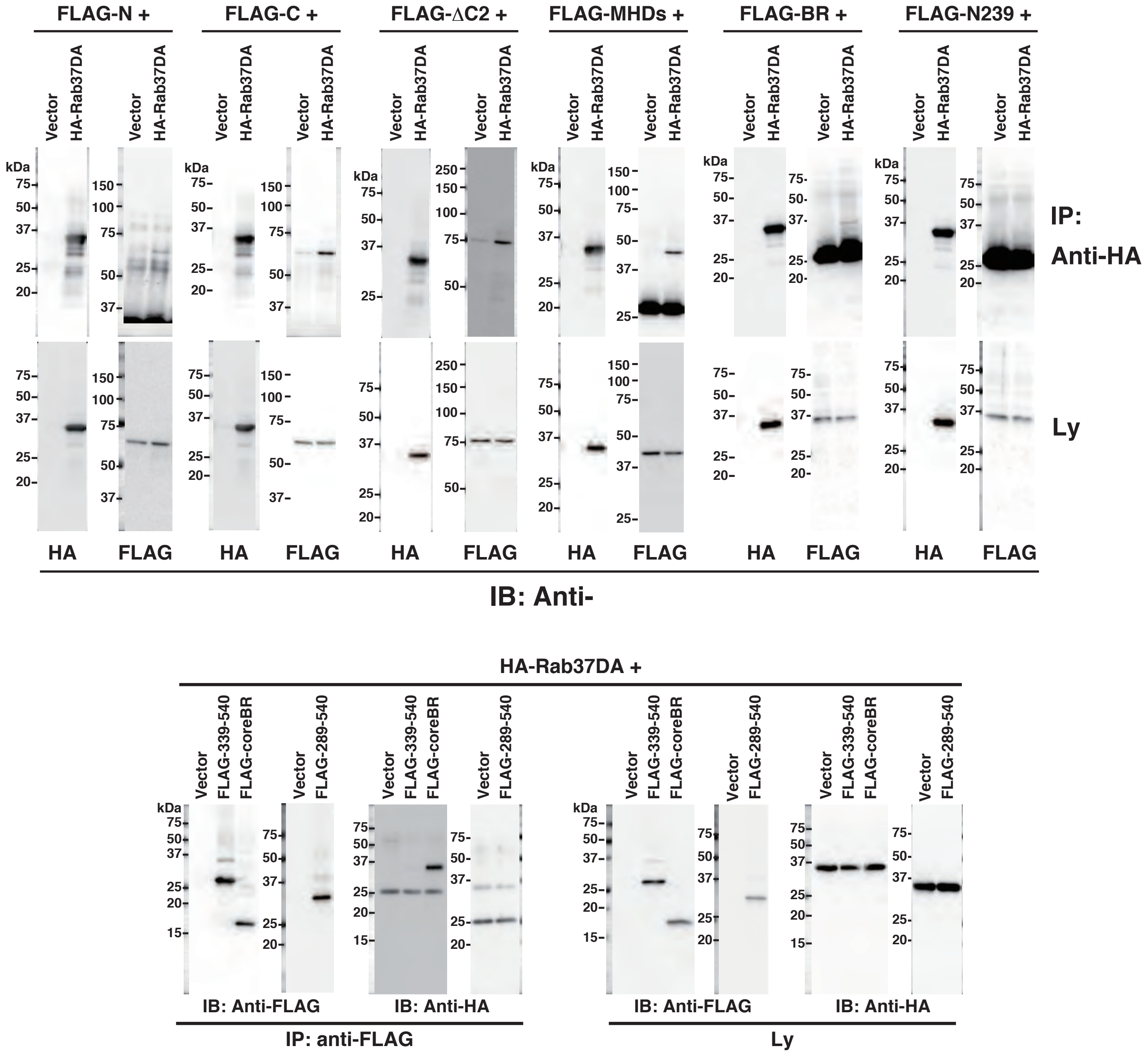
FLAG-N +
FLAG-C +
FLAG-ΔC2 +
FLAG-MHDs +
FLAG-BR +
FLAG-N239 +
Vector
HA-Rab37DA
kDa
IP: Anti-HA
Ly
HA
FLAG
IB: Anti-
HA-Rab37DA +
Vector
FLAG-339-540
FLAG-coreBR
FLAG-289-540
IB: Anti-FLAG
IB: Anti-HA
IP: anti-FLAG
Ly

Fig. S19

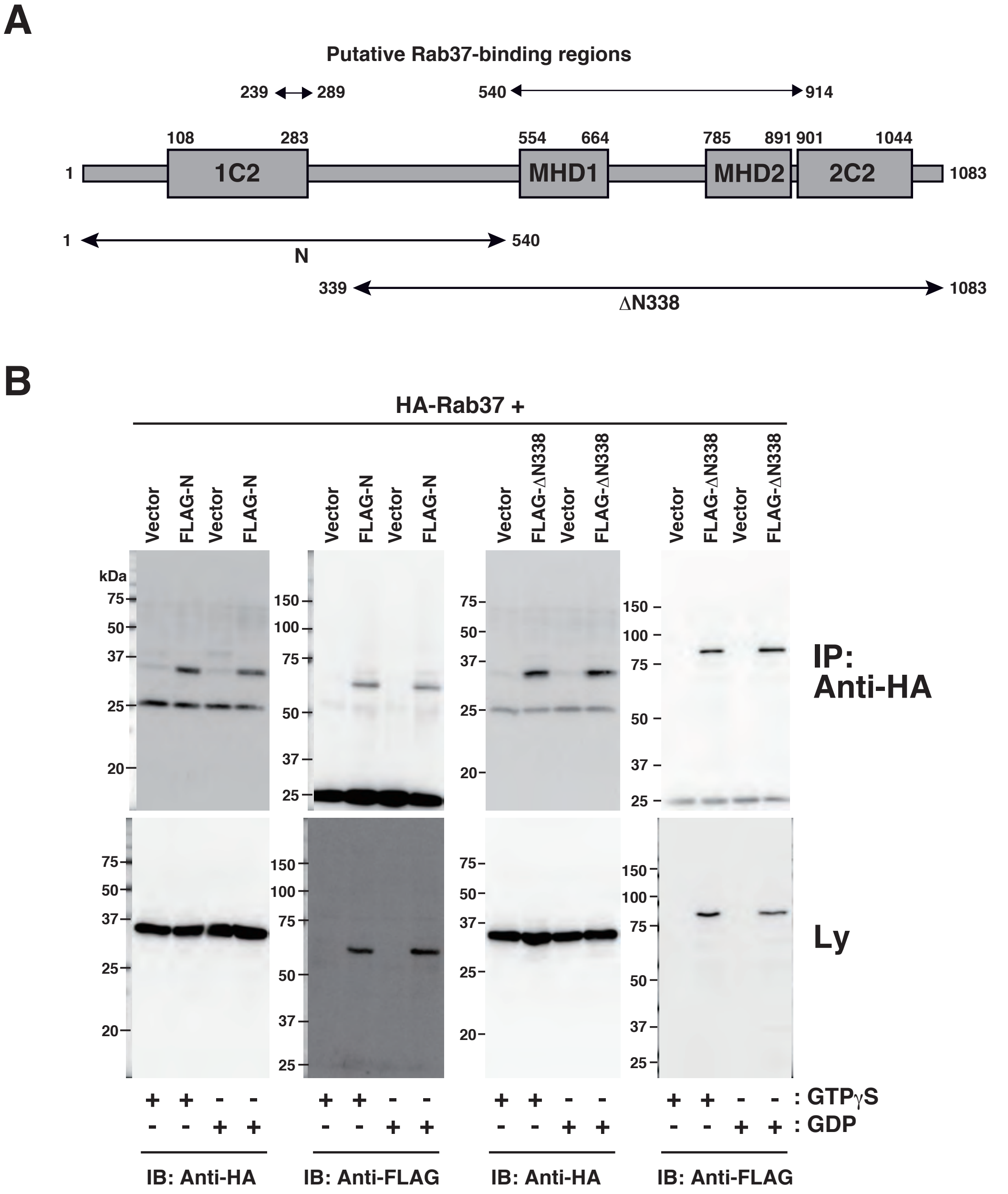

A
Putative Rab37-binding regions
239
289
540
914
108
283
554
664
785
891
901
1044
1
1C2
MHD1
MHD2
2C2
1083
1
540
N
339
1083
ΔN338
B
HA-Rab37 +
Vector
FLAG-N
Vector
FLAG-N
Vector
FLAG-ΔN338
Vector
FLAG-ΔN338
kDa
IP: Anti-HA
Ly
: GTPγS
: GDP
IB: Anti-HA
IB: Anti-FLAG
IB: Anti-HA
IB: Anti-FLAG

Fig. S20

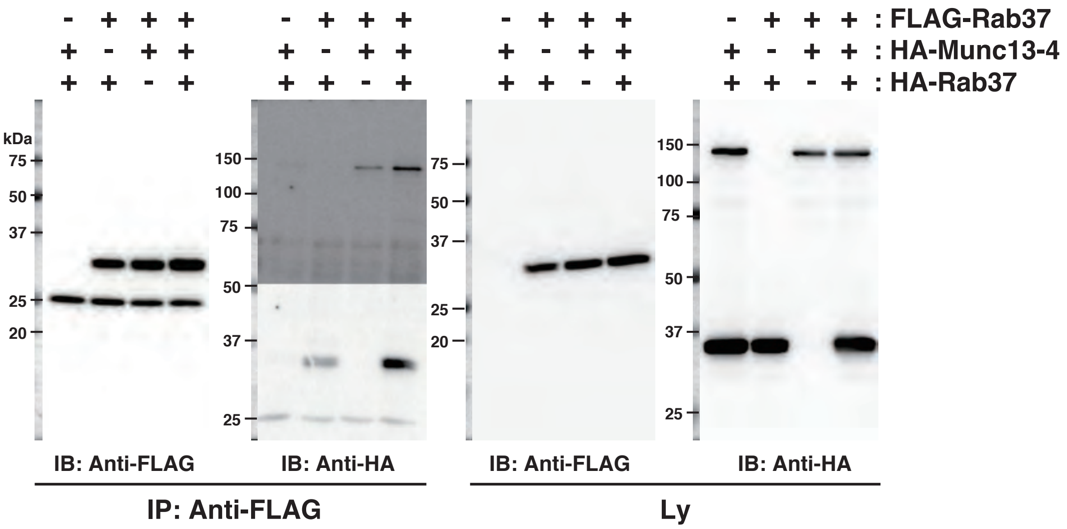

- + + + : FLAG-Rab37
+ - + + : HA-Munc13-4
+ + - + : HA-Rab37
kDa
75
50
37
25
20
150
100
75
50
37
25
75
50
37
25
20
150
100
75
50
37
25
IB: Anti-FLAG
IB: Anti-HA
IB: Anti-FLAG
IB: Anti-HA
IP: Anti-FLAG
Ly

Fig. S21

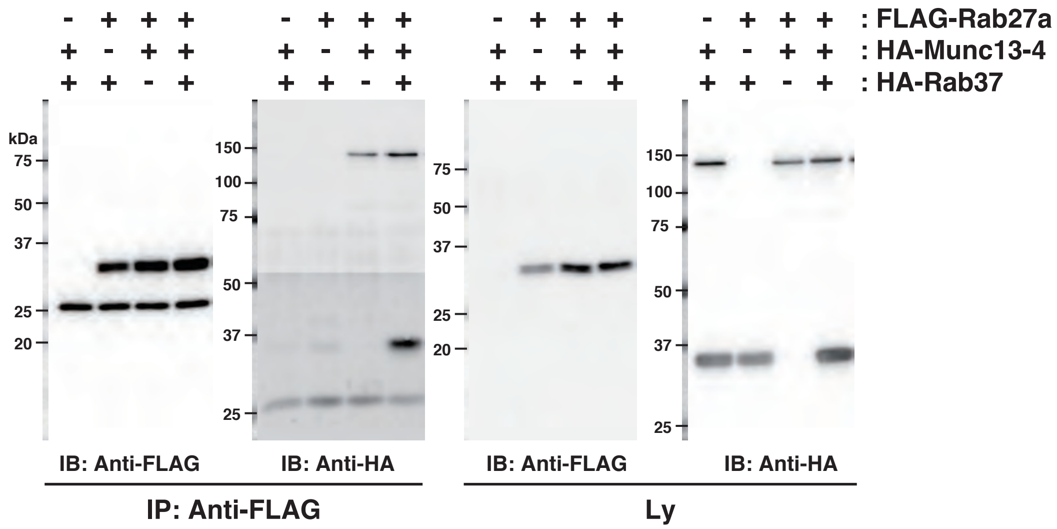

- + + + : FLAG-Rab27a
+ - + + : HA-Munc13-4
+ + - + : HA-Rab37
kDa
75
50
37
25
20
150
100
75
50
37
25
75
50
37
25
20
150
100
75
50
37
25
IB: Anti-FLAG
IB: Anti-HA
IB: Anti-FLAG
IB: Anti-HA
IP: Anti-FLAG
Ly

Fig. S22

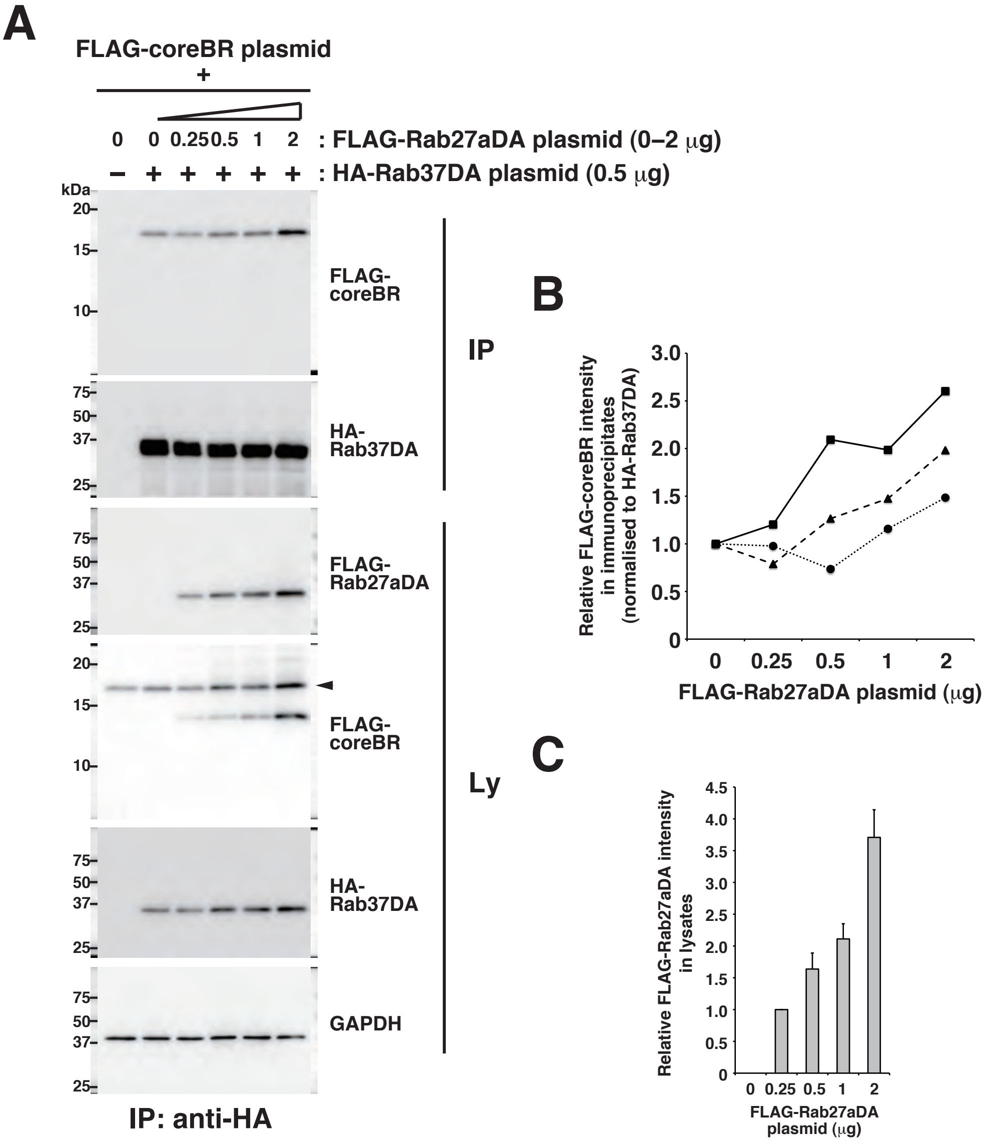
A
FLAG-coreBR plasmid
+
0 0 0.25 0.5 1 2 : FLAG-Rab27aDA plasmid (0–2 μg)
– + + + + + : HA-Rab37DA plasmid (0.5 μg)
kDa
20
15
10
75
50
37
25
FLAG-coreBR
HA-Rab37DA
IP
FLAG-Rab27aDA
FLAG-coreBR
HA-Rab37DA
GAPDH
Ly
IP: anti-HA
B
Relative FLAG-coreBR intensity in immunoprecipitates (normalised to HA-Rab37DA)
3.0
2.5
2.0
1.5
1.0
0.5
0
0 0.25 0.5 1 2
FLAG-Rab27aDA plasmid (μg)
C
Relative FLAG-Rab27aDA intensity in lysates
4.5
4.0
3.5
3.0
2.5
2.0
1.5
1.0
0.5
0
0 0.25 0.5 1 2
FLAG-Rab27aDA plasmid (μg)

Fig. S23

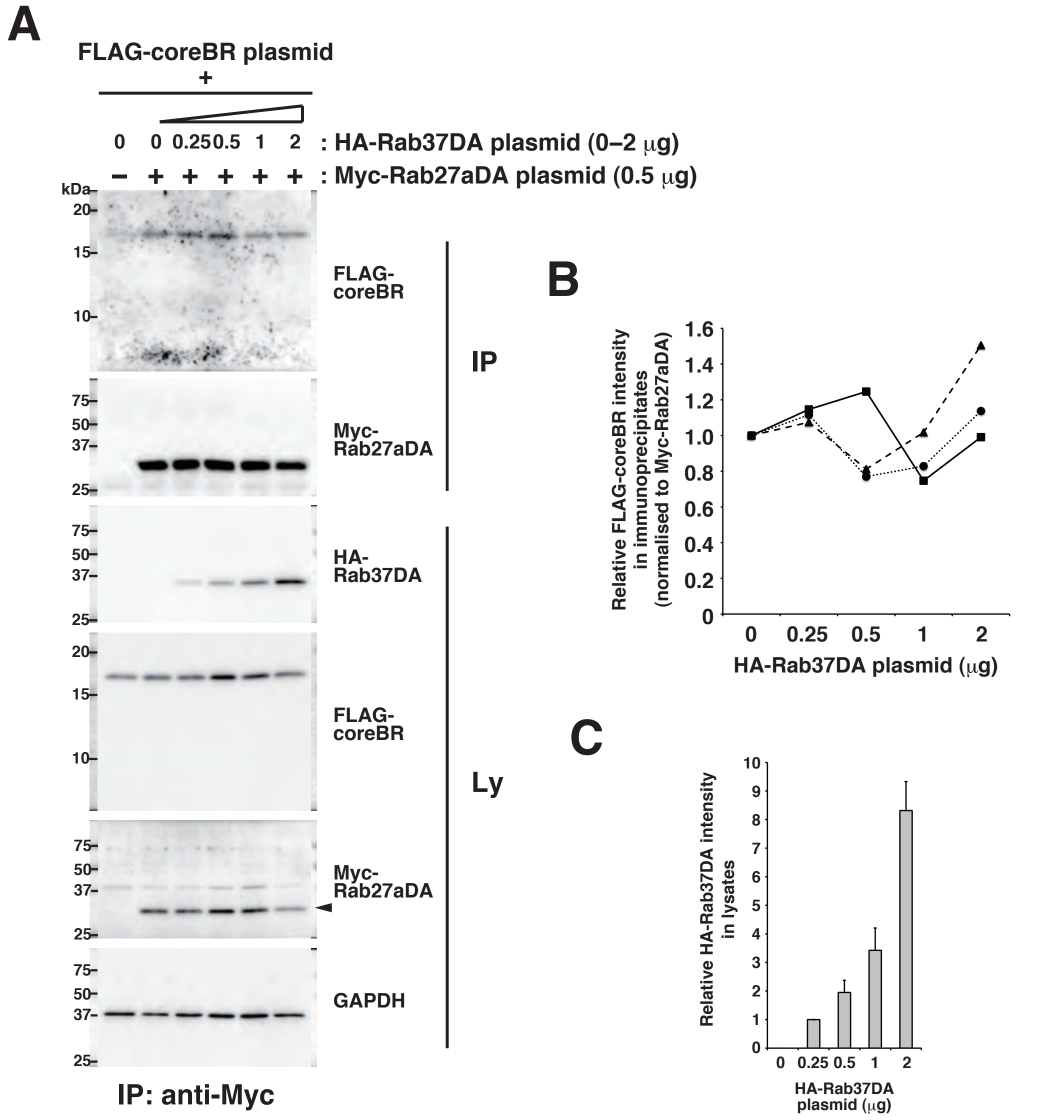

A
FLAG-coreBR plasmid
+
0 0 0.25 0.5 1 2 : HA-Rab37DA plasmid (0–2 μg)
− + + + + + : Myc-Rab27aDA plasmid (0.5 μg)
kDa
FLAG-coreBR
Myc-Rab27aDA
HA-Rab37DA
FLAG-coreBR
Myc-Rab27aDA
GAPDH
IP
Ly
IP: anti-Myc
B
Relative FLAG-coreBR intensity in immunoprecipitates (normalised to Myc-Rab27aDA)
HA-Rab37DA plasmid (μg)
C
Relative HA-Rab37DA intensity in lysates
HA-Rab37DA plasmid (μg)

Fig. S24

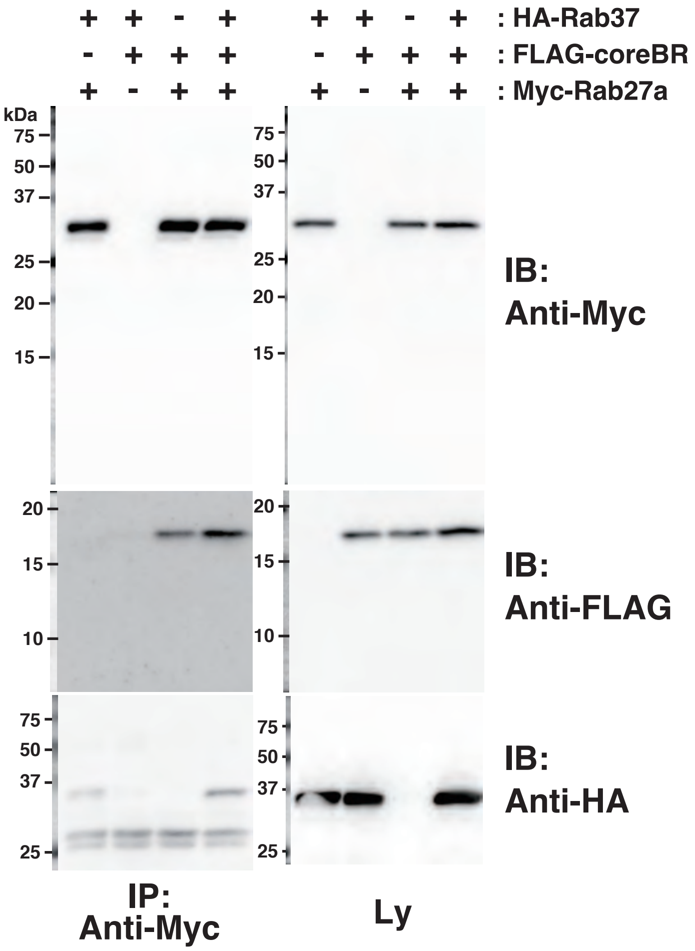

+ + - + + + - + : HA-Rab37
- + + + - + + + : FLAG-coreBR
+ - + + + - + + : Myc-Rab27a
kDa
75
50
37
25
20
15
IB:
Anti-Myc
20
15
10
IB:
Anti-FLAG
75
50
37
25
IB:
Anti-HA
IP:
Anti-Myc
Ly

FIGURE LEGENDS

Figure S1. RT-PCR analyses of the expression of genes (potentially) related to mast cell degranulation in RBL-2H3 cells. Total RNA from the cells was subjected to first-strand cDNA synthesis with (+) or without (−) RT, followed by PCR amplification with specific primer sets for the indicated genes. The expected sizes of the PCR products are indicated.

Figure S2. Expression and intercellular localisation of the Rab37 mutants in RBL-2H3 cells. (A), (B) Expression of the Rab37 proteins in COS7 (A) and RBL-2H3 (B) cells. Lysates (20 μg) prepared from COS7 or RBL-2H3 cells transfected with control (Vector), HA-Rab37, HA-Rab37DA, or HA-Rab37DN plasmid were subjected to western blot analyses (IB) with the antibodies indicated. Arrowheads indicate position of the Rab37 proteins. (C) Intracellular localisation of the Rab37DA protein. RBL-2H3 cells transfected with HA-Rab37DA plasmid were fixed and co-immunostained with anti-HA and anti-CD63 antibodies. Merged image is shown on the right. Bar, 15 μm.

Figure S3. G418 effectively concentrated RBL-2H3 cells carrying the plasmids with a G418 resistance gene Neo^r. (A) Concentration of the EGFP-expressing RBL-2H3 cells by G418. Cells transfected with the EGFP or the control plasmid were cultured in the presence (+G418) or absence (−G418) of G418 for 24 h. The surviving cells were then trypsinised and subjected to flow cytometry. Histograms represent distributions of fluorescence intensity levels in the EGFP (solid line) and the control (dotted line) transfectants. (B) Concentration of the Munc13-4 expressing RBL-2H3 cells by G418. Cells transfected with HA-Munc13-4 (+) or control (−) plasmid were cultured in the presence (+) or the absence (−) of G418 for 24 h. Lysates (20 μg) from the transfected cells were then subjected to western blot analyses (IB) with the antibodies indicated. The graph represents the relative quantity of HA-Munc13-4, with the quantity in G418-untreated cells set to 1. Bars represent mean ± SD (n = 4). **, $p < 0.01$. (C) Effect of G418-mediated concentration of HA-Munc13-4 expressing cells on antigen-induced degranulation in RBL-2H3 cells. The transfected cells described in (B) were sensitised with IgE and stimulated with or without antigen for the indicated time periods. β-hexosaminidase activity was measured in the supernatant and the cell lysate, and the degree of release was expressed as a percentage of the total activity. After the quantity of β-hexosaminidase released without stimulation was subtracted, the mean quantity released from the control transfectant was set to 100. The relative quantity released from the HA-Munc13-4-transfected cells (HA-Munc13-4) is presented as mean ± SD (n = 4). *, $p < 0.05$; **, $p < 0.01$; ***, $p < 0.001$; NS, not significant, compared to the control transfectant.

Figure S4. Efficient knockdown of exogenously expressed Rab37 by its specific siRNA. Lysates (20 μg) of RBL-2H3 cells transfected with Si-control or Si-Rab37 plasmid together with the HA-Rab37 or HA-Rab37* plasmid were subjected to western blot analyses (IB) with

the antibodies indicated.

Figure S5. Endogenous expression of the Rab27 subfamily proteins was unaffected by Rab37 knockdown. Lysates (20 μg) of the G418-surviving RBL-2H3 cells transfected with Si-control or Si-Rab37 plasmid were subjected to western blot analyses (IB) with the antibodies indicated. Arrowheads indicate position of the Rab27 subfamily proteins.

Figure S6. Granular content of Rab37-knockdown RBL-2H3 cells. G418-surviving cells transfected with Si-control or Si-Rab37 plasmid were trypsinised, fixed, permeabilised, immunostained with anti-CD63 antibody, and subjected to flow cytometry. Histograms represent distributions of fluorescence intensity levels of the Si-Rab37 (solid line) and the Si-control (dotted line) transfectants. (B) 1.25×10^5 transfectants described in (A) were lysed and β-hexosaminidase activity was measured in the lysate. The activity of the Si-control transfectant lysate was set to 100 and the relative activity of the Si-Rab37 transfectant lysate is presented as mean ± SD (n = 3). NS, not significant.

Figure S7. LatB disrupts cortical F-actin and enhances both, antigen-induced and spontaneous β-hexosaminidase release from RBL-2H3 cells. (A) Cells were incubated with (+) or without (–) 1 μM LatB, fixed, stained for F-actin with phallacidin, and immunostained with anti-CD63 antibody. Merged images are also shown on the right. Insets represent higher magnification of the boxed region in the merged images. Bar, 30 μm. (B) Cells were sensitised with IgE, incubated with the indicated concentrations of LatB, and then stimulated with (+Ag, grey bar) or without (–Ag, white bar) antigen for 20 min. β-hexosaminidase activity in the supernatant and the cell lysate was measured and the degree of release was expressed as a percentage of the total activity. Bars represent mean ± SD (n = 4). **, $p < 0.01$; ***, $p < 0.001$.

Figure S8. F-actin staining in the Rab37-knockdown RBL-2H3 cells. G418-surviving cells transfected with Si-Rab37 or Si-control plasmid were sensitised with IgE and stimulated with (+Ag) or without (–Ag) antigen in a Ca^{2+}-free medium for 10 min. After fixation, the cells were stained with phallacidin for F-actin and immunostained with anti-CD63 antibody. Merged images are also shown on the right. Insets represent higher magnification of the boxed region in the merged images. Bar, 30 μm.

Figure S9. Evaluation of siRNA-mediated knockdown. (A) Western blot evaluation of siRNA-mediated knockdown. Cell lysate (20 μg) from the G418-surviving RBL-2H3 cells transfected with Si-control plasmid together with Si-control or the following siRNAs (listed in Table S3): Rab27a-specific siRNAs (Si-27a-1, -2, -3), Rab27b-specific siRNAs (Si-27b-1, -2, -3), or Munc13-4-specific siRNAs (Si-13-4-1, -2, -3), were subjected to western blot analyses with the antibodies indicated. Arrowheads indicate positions of the Rab27 subfamily

proteins and Munc13-4. The graphs represent the relative quantity of the Rab27 subfamily proteins or Munc13-4, with their quantity in the Si-control transfectant set to 100. Bars represent mean ± SD (n = 4). *, $p < 0.05$; **, $p < 0.01$, compared to the Si-control transfectant. (B) Degranulation-based evaluation of siRNA-mediated knockdown. The G418-surviving RBL-2H3 transfectants described above were sensitised with IgE and stimulated with (+Ag) or without (−Ag) antigen for 20 min. β-hexosaminidase activity was measured in the supernatant and the cell lysate, and the degree of release was expressed as a percentage of the total activity. Bars represent mean ± SD (n = 4). *, $p < 0.05$; **, $p < 0.01$; NS, not significant, compared to the Si-control transfectant. Based on the results shown in (A) and (B), Si-27a-3, Si-27b-2, and Si-13-4-3 were renamed Si-Rab27a, Si-Rab27b, and Si-Munc13-4, respectively, and subjected to further experiments.

Figure S10. siRNA-mediated knockdown of the Rab27 subfamily proteins. Lysates (20 μg) from the G418-surviving RBL-2H3 cells transfected with Si-control plasmid, together with Si-Rab27a, Si-Rab27b, or Si-control were subjected to western blot analyses (IB) with the antibodies indicated. Arrowheads indicate position of the Rab27 subfamily proteins.

Figure S11. siRNA-mediated downregulation of the Rab27 subfamily mRNA. Total RNA from the G418-surviving RBL-2H3 cells transfected with Si-control plasmid together with Si-Rab27a (n = 3), Si-Rab27b (n = 3), or Si-control (n = 3) were subjected to quantitative real-time RT-PCR. The expression levels of the Rab27 subfamily mRNAs in the Si-control transfectant were set to 100 and their relative expression levels in the cells transfected with their specific siRNAs are presented as mean ± SD (n = 3). ***, $p < 0.001$.

Figure S12. siRNA-mediated knockdown of Munc13-4. Lysates (20 μg) from the G418-surviving RBL-2H3 cells transfected with Si-control plasmid together with Si-Munc13-4 or Si-control were subjected to western blot analyses (IB) with the antibodies indicated. Arrowhead indicates positions of Munc13-4.

Figure S13. Rab37-Munc13-4 interaction. COS7 cells were transfected with HA-Munc13-4 plasmid together with control (Vector), FLAG-Rab37, or FLAG-Rab27a plasmid. Lysates were then prepared in the presence (+) or absence (−) of GTPγS or GDP and subjected to immunoprecipitation with anti-FLAG antibody. The lysates (Ly) and immunoprecipitates (IP) were then subjected to western blot analyses (IB) with the antibodies indicated.

Figure S14. The Rab37-Munc13-4 interaction is GTP-independent. COS7 cells were transfected with FLAG-Munc13-4 plasmid together with control (Vector), HA-Rab37DA, or HA-Rab37DN plasmid. Lysates (Ly) were then prepared and subjected to immunoprecipitation with anti-HA antibody. The lysates (Ly) and immunoprecipitates (IP) were then subjected to western blot analyses (IB) with the antibodies indicated.

Figure S15. The cleavable crosslinker dithiobis succinimidyl propionate (DSP) captures the intracellular Rab-Munc13-4 interactions in RBL-2H3 cells. (A) DSP-dependent detection of the intracellular Rab37-Munc13-4 interaction using exogenously expressed proteins. G418-surviving cells transfected with FLAG-Rab37 (+), HA-Munc13-4 (+), and control (−) plasmid, as indicated, were sensitised with IgE, stimulated with (+) or without (−) antigen for 10 min, and treated with (+DSP) or without (−DSP) DSP before lysis. Lysates were then subjected to immunoprecipitation with anti-FLAG antibody. The lysates (Ly) and immunoprecipitates (IP) were subjected to western blot analyses (IB) with the antibodies indicated. (B) DSP-dependent detection of the intracellular Rab27-Munc13-4 interaction using exogenously expressed proteins. G418-surviving cells transfected with FLAG-Rab27a (+), HA-Munc13-4 (+), and control (−) plasmid, as indicated, were sensitised with IgE, stimulated with (+) or without (−) antigen for 10 min, treated with (+DSP) or without (−DSP) DSP, and then lysed in the presence of GTPγS. Immunoprecipitation and western blot analyses were performed as in (A).

Figure S16. Exogenously expressed Rab37 interacts with endogenous Munc13-4 in RBL-2H3 cells. G418-surviving cells transfected with HA-Rab37 plasmid were treated with DSP, lysed, and subjected to immunoprecipitation with (+) or without (−) anti-Munc13-4 antibody (Anti-Munc13-4 Ab). The lysates (Ly) and immunoprecipitates (IP) were subjected to western blot analyses (IB) with the antibodies indicated. Arrowheads indicate position of HA-Rab37 or Munc13-4.

Figure S17. Rab37 knockdown has little impact on Rab27-Munc13-4 interaction in RBL-2H3 cells. G418-surviving cells transfected with FLAG-Rab27a plasmid with Si-Rab37 or Si-control plasmid was treated with DSP, lysed in the presence of GTPγS, and subjected to immunoprecipitation with (+) or without (−) anti-Munc13-4 antibody (Anti-Munc13-4 Ab). The lysates (Ly) and immunoprecipitates (IP) were subjected to western blot analyses (IB) with the antibodies indicated. Arrowheads indicate position of FLAG-Rab27a or Munc13-4.

Figure S18. Determination of the Rab37-binding regions in Munc13-4. Lysates from COS7 cells transfected with HA-Rab37DA or control (Vector) plasmid, together with the expression plasmid for FLAG-tagged Munc13-4 mutant protein presented in Fig. 6(A) were subjected to immunoprecipitation with anti-HA or anti-FLAG antibody as indicated. The lysates (Ly) and immunoprecipitates (IP) were subjected to western blot analyses (IB) with the antibodies indicated.

Figure S19. GTP-independent Rab37 binding to two binding sites in Munc13-4. (A) Schematic representation of Munc13-4-N and Munc13-4-ΔN338 constructs. Domain organisation of Munc13-4, amino acid positions corresponding to the truncation boundaries of

each construct, and two putative Rab37-binding regions are shown. 1C2: the first Ca^{2+}-binding C2 domain, 2C2: the second Ca^{2+}-binding C2 domain, MHD1: the first Munc13 homology domain, and MHD2: the second Munc13 homology domain. (B) Both, Munc13-4-N and Munc13-4-ΔN338 interact with Rab37 in a GTP-independent manner. Lysates from COS7 cells transfected with HA-Rab37 plasmid, together with control (Vector), FLAG-N, or FLAG-ΔN338 plasmid were prepared in the presence (+) or absence (−) of GTPγS or GDP and subjected to immunoprecipitation with anti-HA antibody. The lysates (Ly) and immunoprecipitates (IP) were subjected to western blot analyses (IB) with the antibodies indicated.

Figure S20. Multiple Rab37 proteins can interact with Munc13-4. COS7 cells were transfected with (+) or without (−) the following plasmids as indicated: FLAG-Rab37, HA-Munc13-4, and HA-Rab37 plasmids. The transfected cells were then lysed and subjected to immunoprecipitation with anti-FLAG antibody. The lysates (Ly) and immunoprecipitates (IP) were subjected to western blot analyses (IB) with the antibodies indicated.

Figure S21. Rab37 interacts with the Rab27-Munc13-4 complex. COS7 cells were transfected with (+) or without (−) the following plasmids as indicated: HA-Rab37, HA-Munc13-4, and FLAG-Rab27a plasmids. The transfected cells were then lysed in the presence of GTPγS and subjected to immunoprecipitation with anti-FLAG antibody. The lysates (Ly) and immunoprecipitates (IP) were subjected to western blot analyses (IB) with the antibodies indicated.

Figure S22. Increasing amount of Rab27 does not interfere with the Rab37-coreBR interaction. (A) Competitive co-immunoprecipitation experiment. COS7 cells were transfected with the FLAG-coreBR plasmid and the indicated amount of FLAG-Rab27aDA plasmid, together with 0.5 μg of HA-Rab37DA (+) or control (−) plasmid, lysed, and subjected to immunoprecipitation with anti-HA antibody. The lysates (Ly) and immunoprecipitates (IP) were subjected to western blot analyses (IB) with anti-HA (3F10), anti-FLAG, and anti-GAPDH antibodies. (B) Relationship between the Rab37-coreBR interaction and amount of the transfected Rab27a plasmid. The graph represents the relative quantity of FLAG-coreBR in immunoprecipitates (normalised to the HA-Rab37DA quantity), with the quantity in the control (0 μg FLAG-Rab27aDA plasmid) transfectant set to 1. Results of three independent experiments are shown. (C) Relationship between expression level of the FLAG-Rab27aDA protein and the amount of the transfected plasmid. The graph represents the relative quantity of FLAG-Rab27aDA in cell lysates, with the quantity in the transfectant with 0.25 μg of the plasmid set to 1. Bars represent mean ± SD ($n = 3$).

Figure S23. Increasing amount of Rab37 does not interfere the Rab27-coreBR interaction. (A) Competitive co-immunoprecipitation experiment. COS7 cells were

transfected with FLAG-coreBR plasmid and the indicated amount of HA-Rab37DA plasmid, together with 0.5 μg of Myc-Rab27aDA (+) or control (−) plasmid, lysed, and subjected to immunoprecipitation with anti-Myc antibody. The lysates (Ly) and immunoprecipitates (IP) were subjected to western blot analyses (IB) with anti-HA (3F10), anti-Myc, anti-FLAG, and anti-GAPDH antibodies. (B) Relationship between the Rab27-coreBR interaction and amount of the transfected Rab37 plasmid. The graph represents the relative quantity of FLAG-coreBR in immunoprecipitates (normalised to the Myc-Rab27aDA quantity), with the quantity in the control (0 μg HA-Rab37DA plasmid) transfectant set to 1. Results of three independent experiments are shown. (C) Relationship between expression level of the HA-Rab37DA protein and the amount of the transfected plasmid. The graph represents the relative quantity of HA-Rab37DA in cell lysates, with the quantity in the transfectant with 0.25 μg of the plasmid set to 1. Bars represent mean ± SD (n = 3).

Fig. S24. Rab37 and Rab27 simultaneously interact with Munc13-4 at the Rab27-binding coreBR region. COS7 cells were transfected with (+) or without (−) the following plasmids as indicated: Myc-Rab27a, FLAG-coreBR, and HA-Rab37 plasmids. The transfected cells were then lysed in the presence of GTPγS and subjected to immunoprecipitation with anti-Myc antibody. The lysates (Ly) and immunoprecipitates (IP) were subjected to western blot analyses (IB) with the antibodies indicated.

Table S1. Primers used for RT-PCR analysis

Gene	GenBank/EMBL/ DDBJ accession No.	Primer	Sequence (5′ −3′)
Rab27a	NM_017317	Forward	ATGTCGGATGGAGATTATGACTAC
		Reverse	TCAACAGCCGCATAACCCCTTCTC
Rab27b	NM_053459	Forward	ATGACTGATGGAGACTATGACTACCTG
		Reverse	CTAGCAGGCACATTTCTTCTCTGC
Rab3d	NM_080580	Forward	ATGGCATCCGCTAGTGAGCCCCCTG
		Reverse	CCTAACAGCCGCAGCTGCTCGGCTG
Munc13-4	NM_138844	Forward	ACAGAGCCATAGCAGCGGAA
		Reverse	GAACATTTCTAGGTGGCAGG
Munc13-3	NM_173146	Forward	CTATTGACGAGAGTGCCTGA
		Reverse	GGTTAGGAACTGGTCAGCAT
Rab37	(NM_021411)	Forward	ATGACTGGCACACCAGGAGCTGCT
		Reverse	TCAGGAGTCACGCAAAGGAGCAGC

Primer set for Rab37 was designed from a rat Rab37 cDNA sequence assembled by comparison between the rat genomic DNA sequence and the mouse Rab37 cDNA (accession No. NM_021411). All primers were assessed for their sequence specificity using BLAST searches (http://blast.ncbi.nlm.nih.gov/Blast.cgi).

Table S2. Primers used for quantitative real-time RT-PCR analyses

Gene	GenBank/EMBL/ DDBJ accession No.	Primer	Sequence (5′ −3′)
Rab27a	NM_017317	Forward	AGACCAGAGGGCTGTGAAAGAG
		Reverse	TGATCAGGTCCAGGAGCATCTC
Rab27b	NM_053459	Forward	AGGGAAGTCAACGAGCGACAAG
		Reverse	CAGAAGGGTTTCCACTGACTTC
GAPDH	BC087743	Forward	ATGACTCTACCCACGGCAAG
		Reverse	CTGGAAGATGGTGATGGGTT
Rab37	(NM_021411)	Forward	TCAGTCCGAACTACGATCTTAC
		Reverse	GGTCACCACTTTATTCCTGAAG

Primer set for Rab37 was designed from a rat Rab37 cDNA sequence assembled by comparison between the rat genomic DNA sequence and the mouse Rab37 cDNA (accession No. NM_021411). All primers were assessed for their sequence specificity using BLAST searches (http://blast.ncbi.nlm.nih.gov/Blast.cgi).

Table S3. siRNAs used for the knockdown of Rab27a, Rab27b, and Munc13-4

Target	GenBank/EMBL/ DDBJ accession No.	siRNA	Sequence (5′ −3′)
Rab27a	NM_017317	Si-27a-1	GGAAGACCAGUGUACUGUACCAGUA
		Si-27a-2	GCAUUCUUCAGGGACGCUAUGGGUU
		Si-27a-3 (Si-Rab27a)	GCCAAGCAAUUGAGAUGCUCCUGGA
Rab27b	NM_053459	Si-27b-1	GGAAAGCCUUUAAGGUACAUCUGCA
		Si-27b-2 (Si-Rab27b)	CACCAGUCAACAGAGUUUCUUGAAU
		Si-27b-3	GAAACCCUUCUGGACUUAAUCAUGA
Munc 13-4	NM_138844	Si-13-4-1	GCCCUGGUCUACUGCAGCCUUAUAA
		Si-13-4-2	ACAUGCUGUGCGUGGUGGUAAAUAA
		Si-13-4-3 (Si-Munc13-4)	CCAGCCCAGCUACACUGUACACUUU

These siRNAs were predesigned by manufacturer and commercially available as Stealth RNAi siRNA^{TM} Duplex oligonucleotides (Thermo Fischer Scientific). After initial assessment of siRNAs by western blot and β-hexosaminidase release analyses (Fig. S9), Si-Rab27a, Si-Rab27b, and Si-Munc13-4 were used for further analyses described in this study.
